# Ionizing Radiation Drives Key Regulators of Antigen Presentation and a Global Expansion of the Immunopeptidome

**DOI:** 10.1016/j.mcpro.2022.100410

**Published:** 2022-09-09

**Authors:** Arun Tailor, Hala Estephan, Robert Parker, Isaac Woodhouse, Majd Abdulghani, Annalisa Nicastri, Keaton Jones, Silvia Salatino, Ruth Muschel, Timothy Humphrey, Amato Giaccia, Nicola Ternette

**Affiliations:** 1 Oxford Cancer Centre for Immuno-Oncology, Nuffield Department of Medicine, University of Oxford, Oxford, United Kingdom; 2 The Jenner Institute, University of Oxford, Oxford, United Kingdom; 3 Oxford Institute of Radiation Oncology, Department of Oncology, University of Oxford, Oxford, United Kingdom; 4 Nuffield Department of Surgical Sciences, John Radcliffe Hospital, Oxford, United Kingdom; 5 Nuffield Department of Medicine, Wellcome Centre for Human Genetics, Oxford, Unitied Kingdom

**Keywords:** AGC, automatic gain control, CT, cancer testis, FDR, false discovery rate, IFN, interferon, PRM, parallel reaction monitoring, PTM, posttranslational modification

## Abstract

Little is known about the pathways regulating MHC antigen presentation and the identity of treatment-specific T cell antigens induced by ionizing radiation. For this reason, we investigated the radiation-specific changes in the colorectal tumor cell proteome. We found an increase in DDX58 and ZBP1 protein expression, two nucleic acid sensing molecules likely involved in induction of the dominant interferon response signature observed after genotoxic insult. We further observed treatment-induced changes in key regulators and effector proteins of the antigen processing and presentation machinery. Differential regulation of MHC allele expression was further driving the presentation of a significantly broader MHC-associated peptidome postirradiation, defining a radiation-specific peptide repertoire. Interestingly, treatment-induced peptides originated predominantly from proteins involved in catecholamine synthesis and metabolic pathways. A nuanced relationship between protein expression and antigen presentation was observed where radiation-induced changes in proteins do not correlate with increased presentation of associated peptides. Finally, we detected an increase in the presentation of a tumor-specific neoantigen derived from Mtch1. This study provides new insights into how radiation enhances antigen processing and presentation that could be suitable for the development of combinatorial therapies. Data are available *via* ProteomeXchange with identifier PXD032003.

## Highlights

• The antigen presentation pathway is induced by radiation in CT26 and MC38.
• A radiation-induced expanded immunopeptidome contains radiation-specific peptides.
• Radiation-specific effects increase in a time-dependent manner.
• Exome sequencing of CT26 reveals a neoantigen which can be induced by radiation.

## Graphical Abstract

**Figure:**
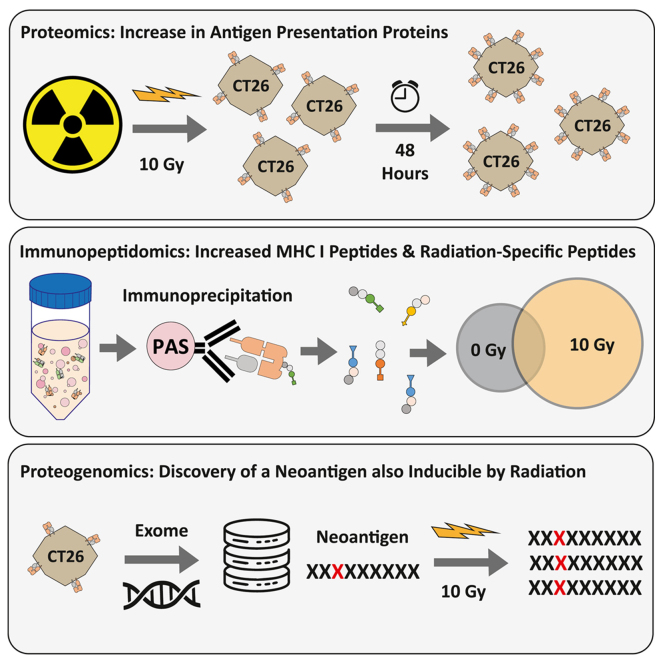

## In-Brief

It has been known that cancer treatment using radiation therapy activates the immune system. Here, we show that radiation increases a signaling component of the immune system called the major histocompatibility complex on tumor cells. This increase makes the tumor more recognizable to T cells whose roles are to specifically find and kill defective or mutated cells. These findings could give physicians a reason to combine radiation therapy with immunotherapies to use the immune effect of radiation for therapeutic benefits.

Data is accumulating that the interactions between radiation therapy and the immune system are beneficial in controlling tumor growth and survival (1, 2). While radiation can have both immuno-stimulatory and immuno-suppressive effects on the tumor microenvironment, major histocompatibility complex (MHC) class I expression is significantly increased on irradiated cells which is essential for the presentation of antigens that will be recognized by CD8+ T-cells (3, 4, 5). The prevailing theory is that the upregulation of MHC class I is largely driven by the induction of type I interferons through cytosolic DNA sensing by the cGAS-STING pathway. However, alternative mechanisms such as a STING-independent NLRC5 pathway have also been proposed (6, 7, 8). While radiation modulates the tumor microenvironment, when administered alone, it fails to produce antitumor immunological responses indicating that a deeper understanding of this complex interplay is required to be able to exploit these changes therapeutically (9).

Antigen presentation requires a highly dynamic process whereby endogenous proteins are continuously digested into peptides, complexed with MHC carrier proteins, and then displayed on the cell surface (10). This immunological process permits the internal proteome of a cell to be sampled for T-cell surveillance. Accordingly, the field of immunopeptidomics aims to isolate and identify these presented peptides using mass spectrometry (11). These methods capture a snapshot of the ‘canonical’ peptides presented at any given time and reflect changes in the proteome occurring under the influence of the cellular environment and treatment conditions. Furthermore, using proteogenomics methods, mutations can be mapped in the cancer genome and tumor-mutation specific, human leukocyte antigen–presented peptides can be identified (12).

In recent years, there has been an increasing interest in the discovery of cancer neoantigens due to their involvement in tumor clearance, making them attractive targets for immunotherapeutic approaches (13). While several neoantigens have been described, the changes in the antigenic landscape including neoantigens induced by treatment with ionizing radiation has not been fully explored. A thorough investigation of the effect of ionizing radiation on antigen presentation will provide insight into its synergy with currently developed, novel immunotherapies such as bispecific antibodies, CAR-T-cells, recombinant T cell receptor and tumor infiltrating leukocyte approaches, and cancer vaccines (10).

We chose the well-characterized murine cancer testis (CT)26 and MC38 cell lines to model the effects of ionizing radiation on changes in the proteome and MHC-presented immunopeptidome in colorectal cancer cells to investigate the pathways driving MHC class I upregulation, which could be translated from pre-clinical studies to human clinical trials (14). Furthermore, by using a quantitative proteomics approach, we explored how other proteins are regulated upon irradiation, specifically those upstream of antigen processing. The paired quantitative analysis of the CT26 cell line using proteomics and immunopeptidomics provides a powerful approach to understand how radiation drives key regulators of antigen presentation and how these changes are reflected in the overall regulation of all peptides in the immunopeptidome.

## Experimental Procedures

### Cell Culture & Irradiation

CT26 WT Cells (ATCC - CRL-2638) were grown in RPMI (Gibco) supplemented with 10% fetal bovine serum (Gibco) and 1% Penicillin-Streptomycin solution (Sigma) at 37 °C, 5% CO_2_. Cells were grown to 70% confluence and media was changed immediately before irradiation. Cells were irradiated with a single acute dose of 10 Gy of Caesium (^137^Cs) gamma rays at a dose rate of 0.624 Gy/min using a GSR D1 Gsm (Gamma-Service Medical GmbH). Cells were returned to 37 °C, 5% CO_2_ conditions for 0 h, 24 h, 48 h, or 72 h for proteomics experiments and for 24 h and 48 h for immunopeptidomics experiments. Upon harvesting, cells were washed in DPBS (Gibco) and gently scraped to release cells before counting, pelleting, and flash freezing on liquid nitrogen. One T75 flask of cells was harvested per condition for proteomics experiments and a final count of 300 × 10^6^ cells per condition for immunopeptidomics experiments. The same conditions were used for experiments with the cell line MC38 (CVCL_B288) except cells were not treated for immunopeptidomics at the 48 h time point.

### Lysate Preparation

Frozen cell pellets were lysed in 3 ml lysis buffer (1% IGEPAL 630, 100 mM Tris pH 8.0, 300 mM NaCl supplemented with complete Protease Inhibitor Cocktail, EDTA-free, Roche) by pipetting mildly up and down and incubating end-over-end at 4 °C. Lysates were then cleared by sequential centrifugation at 4 °C at first 500*g* and then 21,000*g* for 10 min and 1 h, respectively.

### Proteomics Sample Preparation

Cleared lysates were normalized to 15 μg per sample using the bicinchoninic acid Protein Assay (Peirce) and adjusted to 5% SDS in a final volume of 20 μl sample. Samples were reduced with 10 mM DTT for 15 min followed by alkylation with 55 mM iodoacetamide for a further 15 min and a repeated addition of 10 mM DTT. The reduced and alkylated samples in 25 μl underwent digestion using the S-Trap midi protocol according to the manufacturer’s instructions. Briefly, samples were acidified in 2.5% phosphoric acid and dissolved in 165 μl of 100 mM TEAB (triethylammonium bicarbonate) in 90% MeOH. Samples were transferred to S-Traps and centrifuged at 4000*g* for 30 s. Samples were washed 3 times in 100 mM TEAB/90% MeOH followed by the addition of 1.5 μg of trypsin (NEB) dissolved in 20 μl 50 mM TEAB and incubated for 2 h at 47 °C. Finally, samples were eluted in 40 μl 50 mM TEAB followed by 40 μl 0.2% formic acid followed by 40 μl 50% acetonitrile. Eluents were pooled and dried until ready for mass spectrometric acquisition.

### Preparation of MHC Class I Immunoresin

MHC Class I antibody immunoresin for each biological replicate for CT26 was prepared by crosslinking 5 mg of antibody clone 34.1.2s (recognizing H-2-Kd, Dd, and Ld, purified from hybridoma cells, ATCC HB79) to 0.5 ml of Sepharose protein A bead slurry in 10 column volumes (cv) of 40 mM dimethyl pimelimidate in borate buffer, pH 8.3 for 30 min at room temperature. The reaction was stopped with 10 cv of ice-cold 0.2 M Tris pH 8.0, followed by a washing step of 10 cv of 0.1 M citrate (pH 3.0) to remove unbound antibody, and the column was equilibrated with 10 cv of 50 mM Tris (pH 8.0). The same procedure was performed for the immunoaffinity capture of MHC molecules in MC38 except the antibody clone 28-8-6S was used (recognizing H-2-Db and Kb, purified from hybridoma cells, ATCC HB51).

### MHC Peptide Enrichment & Purification

Cleared lysates were incubated with immunoresin overnight at 4 °C under mild agitation. Columns were washed using 10 cv of 50 mM Tris pH 8.0 containing 150 mM NaCl, 450 mM NaCl, and a final wash with no salt. Peptide MHC complexes were eluted with the addition of 5 cv of 10% acetic acid. Samples were dried and resuspended in 0.1% TFA, 1% acetonitrile in water, and loaded onto a monolithic column (4.6 × 50 mm ProSwift RP-1S, ThermoFisher Scientific) on a preparative Ultimate 3000 HPLC system (Thermo Scientific). Peptides were separated from larger complex components by applying a 10-min gradient from 2 to 35% buffer B (0.1% TFA in acetonitrile) with a flow rate of 1000 μl/min. Concatenated fractions, excluding larger protein containing fractions, were pooled and dried.

### LC-tandem Mass Spectrometry

Peptides were dissolved in loading solvent (0.1% (v/v) TFA, 1% (v/v) Acetonitrile) and analyzed by a Q Exactive HF-X mass spectrometer coupled to an Ultimate 3000 RSLCnano System (Thermo Scientific). Initially, peptides were loaded onto the trap column in loading solvent by an Acclaim PepMap 100 C18 5 μM 0.1 × 20 mm column before analytical separation by a 60 min linear acetonitrile gradient of either 2 to 25% for immunopeptidomics or 2 to 35% for proteomic samples in water containing 1% (v/v) DMSO and 0.1% (v/v) formic acid at a flow rate of 250 nl/min on a 75 μm × 50 cm PepMap RSLC C18 EasySpray column at 40 °C (Thermo Scientific). An EasySpray source was used to ionize peptides at 2 kV with an ion transfer capillary temperature of 305 °C and Funnel RF level of 40. For immunopeptidomics samples, data-dependent acquisition consisted of a 320 to 1600 m/z full-MS scan (120,000 resolution, 100 ms accumulation time, target automatic gain control (AGC) 3 × 10^6^) and 20 dependent MS^2^ scans (60,000 resolution, 120 ms accumulation time, target AGC 5 × 10^5^). For proteomic samples, data-dependent acquisition was adapted from a standard 15 Hz proteomics duty cycle 320 to 1600 m/z full-MS scan (60,000 resolution, 45 ms accumulation time, target AGC 3 × 10^6^) and 12 dependent MS^2^ scans (30,000 resolution, 54 ms accumulation time, AGC 2 × 10^5^). The quadruple isolation width was 1.6 m/z (Immunopeptidomics) or 1.3 m/z (Proteomics) and only 2 to 4 charge states were fragmented with a normalized high energy collisional dissociation energy set to 25% for immunopeptidomics and 28% for proteomic samples. Dynamic exclusion was set for 30s and all data were acquired in profile mode.

### Qualitative Mass Spectrometry Data Analysis

MS data were analyzed with Peaks v10.0 (Bioinformatics Solutions) for the identification of peptide sequences matching to databases generated by integration of exome-identified protein variants and all reviewed mouse SwissProt protein entries (17,027 Protein Entries, Downloaded 23/12/2019). For CT26 analyses, irrelevant MHC class I alleles were removed from the SwissProt database to retain only H-2-Kd, H-2-Dd, and H-2-Ld which are known to be present in BALB/c mice. Searches were performed with the following parameters: no enzyme specificity, no peptide modifications, peptide tolerance: ± 5 ppm, and fragment tolerance: ± 0.03 Da. The results were filtered using a peptide-level false discovery rate (FDR) of 1% established through parallel decoy database searches. For the PEAKS PTM search, all 313 built-in modifications were selected for analysis. Data was also searched in a separate analysis to include common variable modifications including deamidation (NQ), cysteinylation, methionine oxidation, and protein n-terminal acetylation.

### Analysis of Differential Expression

For quantitative analysis, the data was analyzed by Progenesis QI for proteomics (Waters) for chromatographic alignment, normalization, and determination of individual peptide ion abundances. Label free quantification was performed through a calculation of area-based abundance on the top three unique peptides per protein. The method for normalization was scalar factor normalization to all proteins. All proteins which were quantifiable by progenesis were included for analysis (supplemental Table S6). A two-way ANOVA analysis was applied to assess significant regulation of peptides between irradiated and control conditions across time points. Normalized protein quantification data for 4150 quantifiable proteins was exported from progenesis, and differential expression analysis was performed using the Differential Expression of Proteins package (R version 3.6.3). Data imputation was performed using the maximum likelihood estimation method (maximum likelihood-based imputation). Volcano plots for each time point depicting -Log_10_ *p* values (limma) against Log_2_ Fold Change showed significantly downregulated and upregulated proteins defined by a -Log_10_*P*cutoff of 5 and a Log_2_ Fold Change cutoff of 1 (15). The same pipeline was performed for the quantitative analysis of immunopeptidomics data, except, label free quantification was based on all associated peptides per protein.

### Pathways Analysis

Pathway analysis was performed with Ingenuity Pathway Analysis (Qiagen). Differential expression data generated from the Differential Expression of Proteins analysis was inputted into the Ingenuity pipeline. A standard cut-off of 0.58 for LogFC which correlates to a FC of 1.5 in either direction and a *p*-value < 0.05 was used for all analyses. The species was set to mouse for all analyses. Overrepresentation or ‘expression’ data used a Log FC of 0.58, which correlates to a FC of 1.5 only based on increased fold change.

### Western Blot Analysis

Cells were collected and lysed in UTB (9 M urea, 75 mM Tris–HCl pH 7.5, 0.15 M β-mercaptoethanol) and briefly sonicated. Primary antibodies were LMP2 (ab3328, abcam), LMP7 (13635S, Cell Signaling), TAP2 (PA5-37414, Thermo Fisher), and β-actin (sc-69876 Santa-Cruz biotechnology). Secondary antibodies were IRDye680RD Goat anti-Mouse IgG (H + L) and IRDye 800CW Donkey anti-Rabbit IgG (H + L) from LI-COR Biosciences. Odyssey IR imaging technology (LI-COR Biosciences) was used for imaging.

### Flow Cytometry

Cells were harvested and fixed with 4% paraformaldehyde for 10 min, then cells were incubated with primary antibody clone 34.1.2s (recognizing H-2-Kd, Dd, and Ld, purified from hybridoma cells, ATCC HB79) for 20 min followed by an incubation with secondary antibody Alexa-Fluor 488 goat anti-mouse (A11017, Invitrogen) for 10 min. Samples were run on a Cytoflex flow cytometer (Beckman Coulter, Life sciences) and data were analyzed using FlowJo software (www.flowjo.com).

### DNA Isolation and Sequencing

DNA was isolated from untreated CT26 cells using the DNeasy Blood & Tissue (Qiagen) isolation kit as per the manufacturer’s instructions. The qualified genomic DNA sample was randomly fragmented by Covaris technology, and the size of the library fragments was mainly distributed between 150 bp and 250 bp. The end repair of DNA fragments was performed, and an "A" base was added at the 3′-end of each strand. Adapters were then ligated to both ends of the end repaired/dA–tailed DNA fragments for amplification and sequencing. Size-selected DNA fragments were amplified by ligation-mediated PCR, purified, and hybridized to the BGI exome array for enrichment. Nonhybridized fragments were then washed out and captured products were circularized. The rolling circle amplification was performed to produce DNA Nanoballs. Each resulting qualified captured library was then loaded on BGISEQ-500 sequencing platforms, and we performed high-throughput sequencing for each captured library to ensure that each sample met the desired average sequencing coverage.

### Exome Sequencing Analysis

The Nextflow (v20.10.0) nf-core pipeline Sarek (v2.7) performed data processing and variant calling (16, 17, 18). In short, the pipeline begins by mapping fastq-formatted read data to the reference genome GRCm38 using bwa-mem (v2.0), applies GATK (v4.1.7.0) to mark duplicates and perform base recalibration, uses tools Manta (v1.6.0) and Strelka (v2.9.10) for variant calling, and finally MultiQC (v1.8) summarizes all recorded metrics for quality control. At this stage, output files were filtered to retain variants marked “PASS” by Strelka’s filtering standards. Additional filtering set a threshold mutation read coverage of at least 10 reads (DP vcf field for SNVs, DPI for indels) (19, 20, 21, 22, 23).

An in-house–modified version of neoantigen prediction tool MuPeXI, known as TUNAPASTA, was used to further process these variants to create a sample-specific protein database (24). As part of the modified processes, variant annotation was performed by Ensembl-VEP (v101.0) (25). Mutations in transcripts identified as other than “protein-coding” gene biotype were excluded, as were any transcripts that included the biotype “NMD_mediated_decay”. Ensembl-VEP also provided global allele frequency information, used in the subsequent step of analysis. TUNAPASTA then generated output files including this additional collated information, along with customized short protein sequences (desired length 31 aa) and fasta file outputs with paired normal and mutant sequences. The key modifications in TUNAPASTA most relevant to this project were the tweaking of input format requirements to accept Strelka-generated vcf files, as well as the generation of the described additional outputs necessitated by the workflow.

Downstream processing in R was used to further filter and prepare the fasta file. Normal and mutant peptide sequences are split into separate paired entries. To emulate the nature of somatic variants being present in less than 1% of surveyed populations, mutations were retained at this threshold according to the “highest allele frequency observed in any population from 1000 genomes, ESP, or gnomAD” projects. This final stage of filtering generated the provided fasta files of peptides representing the contextual protein sequences of likely somatic mutations.

### Quantitative Assessment of Mutated Peptides

Heavy-labeled peptides with a modified lysine residue at the first position K(L-(13C6,15N2)-Lysine)YLSVQSQL and K(L-(13C6,15N2)-Lysine)YLSVQGQL along with light peptides KYLSVQSQL and KYLSVQGQL were ordered from Mimotopes Pty Ltd at 97% purity and were dissolved in loading buffer for analysis. To determine the quantitative range for each peptide, different amounts (0, 5, 10, 25, 50, 100, and 150 fmol) of light peptide were mixed with a constant amount (100 fmol) of heavy peptide. An elastase digest of HeLa cells has been previously used as an LC/MS quality control for immunopeptidomics experiments to replicate the nontryptic nature of MHC peptides (26). Accordingly, 100 ng of the elastase digest was used as a background matrix in each standard to replicate a similar total ion density as the samples, and after analysis, the ratio of light/heavy was used to generate a standard curve. The heavy peptide mix was spiked into all samples and standards at a final concentration of 100 fmol. The parallel reaction monitoring (PRM) method was run at a resolution of 30,000, an AGC target of 3e^6^, and maximum IT of 100 ms. Fourteen transitions were added to the inclusion criteria in the PRM method which included 10 additional transitions of peptides which were not used in this study. An isolation window of 1.4 m/z was chosen and a (N)CE of 19 was chosen for all four peptides with a 15-min window with a start time of 35.56 min and an end time of 50.56 min. The same chromatography conditions were applied as per all immunopeptidomics experiments detailed above. All peptide transitions were optimized for (N)CE and retention time, and quantitation of endogenous peptides and standard curve was performed using Skyline Version 21.1.0.278. Data has been uploaded to Panorama Public at the following url: https://panoramaweb.org/ct26_mtch1_quant.url

### Experimental Design & Statistical Rationale

Proteomics experiments were performed in triplicate biological replicates with duplicate technical injections to observe fine changes in the proteome. Technical injections were averaged before proceeding with differential expression analysis. Strong alignment scores were observed for these samples (above 95%) and therefore maximum likelihood estimation was deemed the most appropriate statistical method for imputing missing values. Furthermore, a time-matched control condition was included to accurately assess the dose-time relationship *via* a two-way-ANOVA. Immunopeptidomics samples were performed in triplicate biological replicates to ensure all sample acquisition could take place within one LC/MS instrument calibration cycle. Immunopeptidomics data was assessed through the concatenated injection of odd and even fractions to maximize the total number of peptides to focus on antigen discovery and radiation-specific changes. To improve clarity for the reader, quantitative assessments of the global immunopeptidome have been shown as both total number of unique peptides and summed peptide intensity. Data was filtered to a stringent 1% peptide-level FDR across samples to account for only the most confident peptide-spectrum matches to be carried forward for downstream analysis in both immunopeptidomics and proteomics analysis. The expansion of the CT26 control samples included several additional samples, which were run previously under the same method to expand the total comparative pool. This enabled us to add further confidence to peptides, which were specific to the radiation condition. *p*-values for all figures have been denoted by ∗ <0.05, ∗∗ <0.01, ∗∗∗ <0.001, except for interaction *p*-values for the two-way-ANOVA conducted for the time-course proteomics analysis for which numerical values have been used.

## Results

### Proteomics Analysis Reveals Time-dependent Regulation of Protein Clusters upon Irradiation

**Fig 1:**
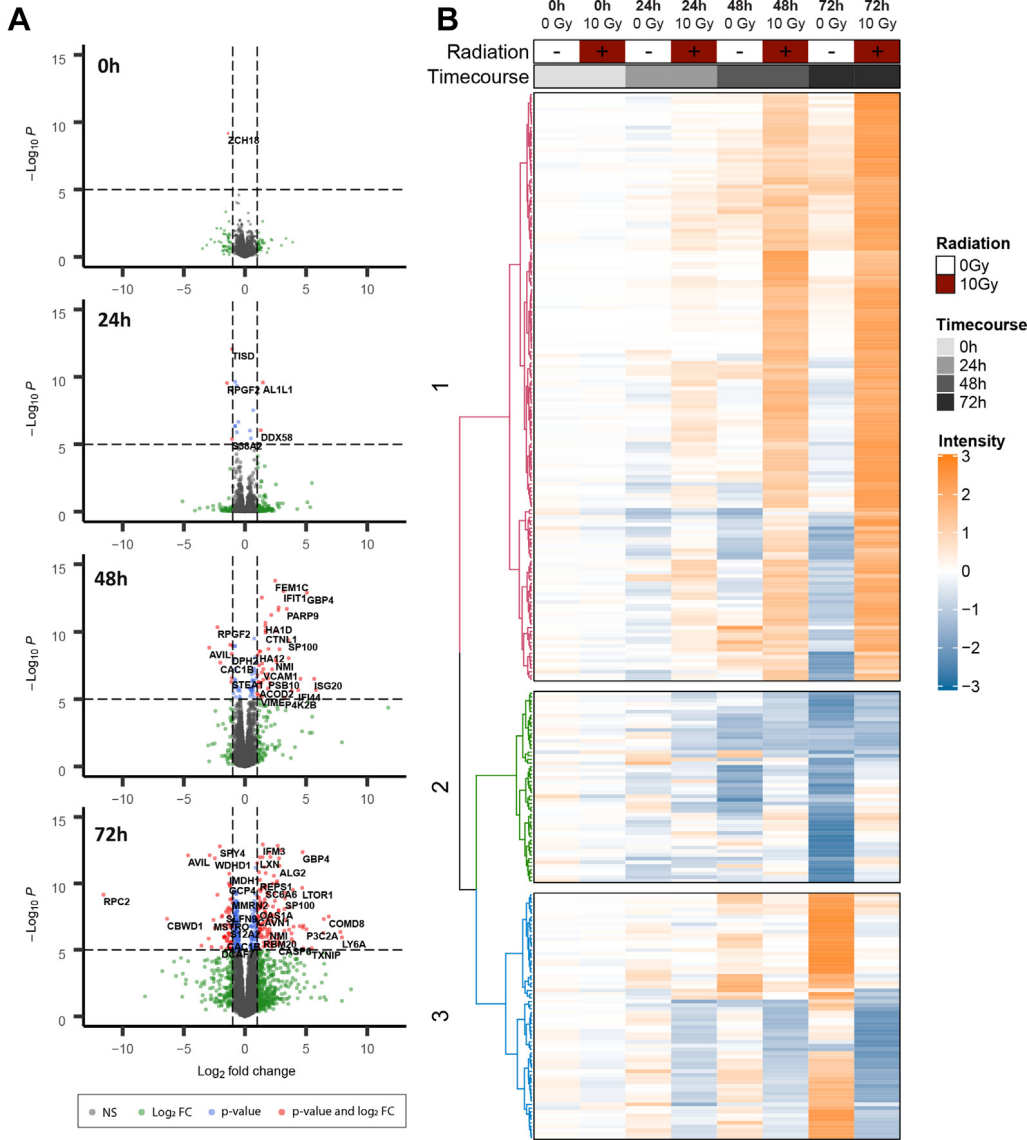
**Differential expression of CT26 proteome at 0 h, 24 h, 48 h, and 72 h post single dose 10 Gy irradiation.***A*, volcano plots of differentially expressed proteins (*red*) isolated by time point (limma, -Log10*p* /> 5, -Log2 fold change >1.5). *B*, heatmap depicting deregulated proteins with two-way-ANOVA interaction *p* value less than 0.0001 (279 proteins). CT, cancer testis.

Our first objective was to explore the overall changes in protein expression at different time points following a single dose of radiation to define the optimal treatment time for larger scale immunopeptidomics experiments. Accordingly, cells were gamma-irradiated at doses of 0 Gy and 10 Gy and were harvested at 0-, 24-, 48-, and 72-h following treatment. A label-free quantitative proteomics experiment was performed to assess the differential expression of 4150 quantifiable proteins at each time point for radiation-induced changes. Importantly, samples were lysed to enrich for the cytoplasmic proteome as opposed to the nuclear proteome. Differentially expressed proteins were significantly increased after irradiation in a time-dependent manner and were skewed toward upregulation (time postirradiation, downregulated proteins, upregulated proteins; 0 h, 1↓, 0↑; 24 h, 3↓, 3↑; 48 h, 7↓, 45↑; 72 h, 54↓, 125↑) (Fig. 1*A*). To perform a global analysis of the change in protein expression over time, proteins were ranked by significance based on a two-way-ANOVA to assess the relationship between treatment and time postirradiation. Proteins with an interaction *p* value less than 0.0001 (279 proteins) were clustered in a heatmap to demonstrate proteins which had a time-dependent increase (cluster 1) or decrease (cluster 3) in protein expression (Fig. 1*B*). The second cluster represents proteins with increased expression over time but only in untreated samples. A clear time-dependent trend was observed in both directions suggesting that radiation-induced changes in the proteome significantly occur with time postirradiation.

A principal component analysis of the data showed clear groupings for most of the biological replicates with distance (greater variation) between the groups increasing over time (supplemental Fig. S1). We did observe an increasing variability in the control samples that may have been caused by confluence over increasing time points, however, this was not observed in the irradiated samples. Cell density may partially explain the changes in proteins in the control cells that were unaltered in irradiated cells (cluster 2 in the heatmap, Fig. 1*B*). To avoid any such effects, we decided to perform the analysis of the immunopeptidome at the 24- and 48-h time points only.

### Radiation-Induced Changes are Likely Driven by Pattern Recognition Receptor Sensing and Interferon Signaling

**Fig 2:**
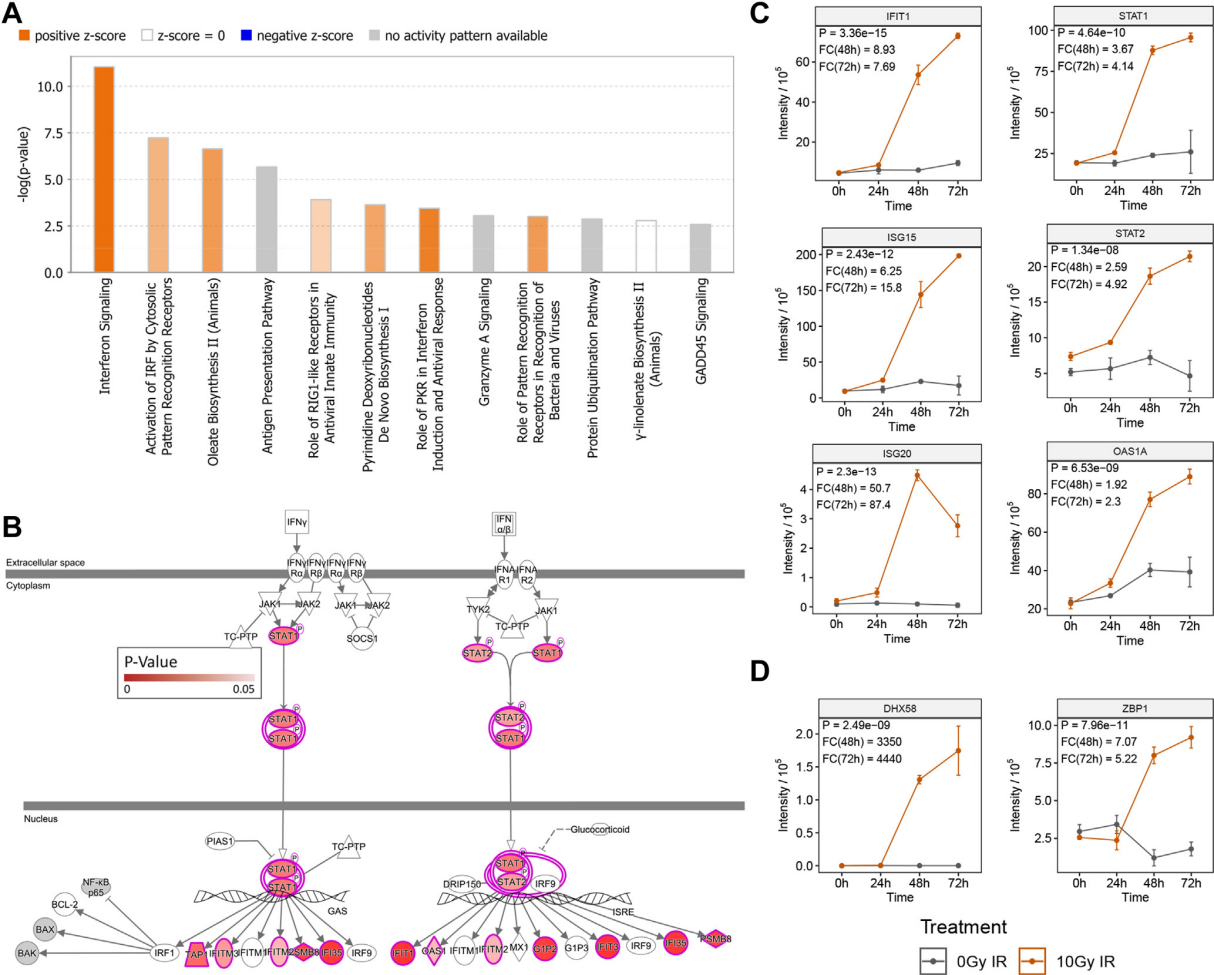
**Overall pathway analysis and interferon signaling in the CT26 proteome upon irradiation.***A*, Ingenuity Pathway Analysis of differentially expressed proteins at 48 h post 10 Gy irradiation (-Log2 fold change >1.5). *B*, Interferon Signaling Ingenuity Pathway visualized with *p*-values for quantifiable proteins in the CT26 proteome. *C*, normalized intensity plots of proteins encompassing the Interferon Signaling Pathway. *D,* the Activation of IRF pathway in the CT26 proteome upon irradiation. Points are representative of mean ± SD values of three biological replicates and *p*-values are representative of two-way ANOVA, where *gray* indicates untreated, and *orange* indicates 10 Gy treated. CT, cancer testis.

To explore the nature of radiation-induced changes in the CT26 proteome, we used the Ingenuity Pathway Analysis software and evaluated changes in expressed proteins at the 48-h time point (*p* > 0.05, FC > 1.5). The top 12 canonical pathways with a -log(*p*-value) greater than 1.5 were graphically represented showing positive (orange) or negative (blue) z-scores according to upregulated or downregulated pathways, respectively (Fig. 2*A*). Pathways with no activity pattern are shown in gray, where a z-score could not be discerned by the software. The top two upregulated pathways were “Interferon Signaling” (-logP = 11) and “Activation of IRF by Cytosolic Pattern Recognition Receptors”(-logP = 7.23).

The "Interferon Signaling" pathway has been mapped in Figure 2*B*, showing the encompassing proteins colored by their *p*-value. A series of interferon-inducible proteins were shown to be upregulated including ISG15 (*p* = 2.43e-12; FC-48 h = 6.25), ISG20 (*p* = 2.3e-13; FC-48 h = 50.7), IFIT1 (*p* = 3.35e-15; FC-48 h = 8.93), and OAS1 (*p*= 6.53e-09; FC-48 h = 1.92) along with upstream signaling components STAT1 (*p* = 4.64e-10; FC-48 h = 3.67) and STAT2 (*p* = 1.34e-08; FC-48 h = 2.59) (Fig. 2*C*). Importantly, two of the most significantly altered proteins in this dataset included DNA-sensing proteins ZBP1 also known as DAI (*p* = 7.96e-11; FC-48 h = 7.07) and DHX58 (*p* = 2.49e-09; FC-48 h = 3350), which are at the center of the “Activation of IRF by cytosolic Pattern Recognition Receptors” pathway (Figs. 2*D* and S2), suggesting a role for these molecules in cytosolic DNA sensing and subsequent IRF3 and 7 activation that could lead to the observed strong type I interferon (IFN) response. Significant changes in important proteins encompassing the Interferon Signaling pathway support the relevance of this pathway in the radiation response.

### Irradiation Alters the Cellular Profile for Protein Turnover and Antigen Presentation

**Fig 3:**
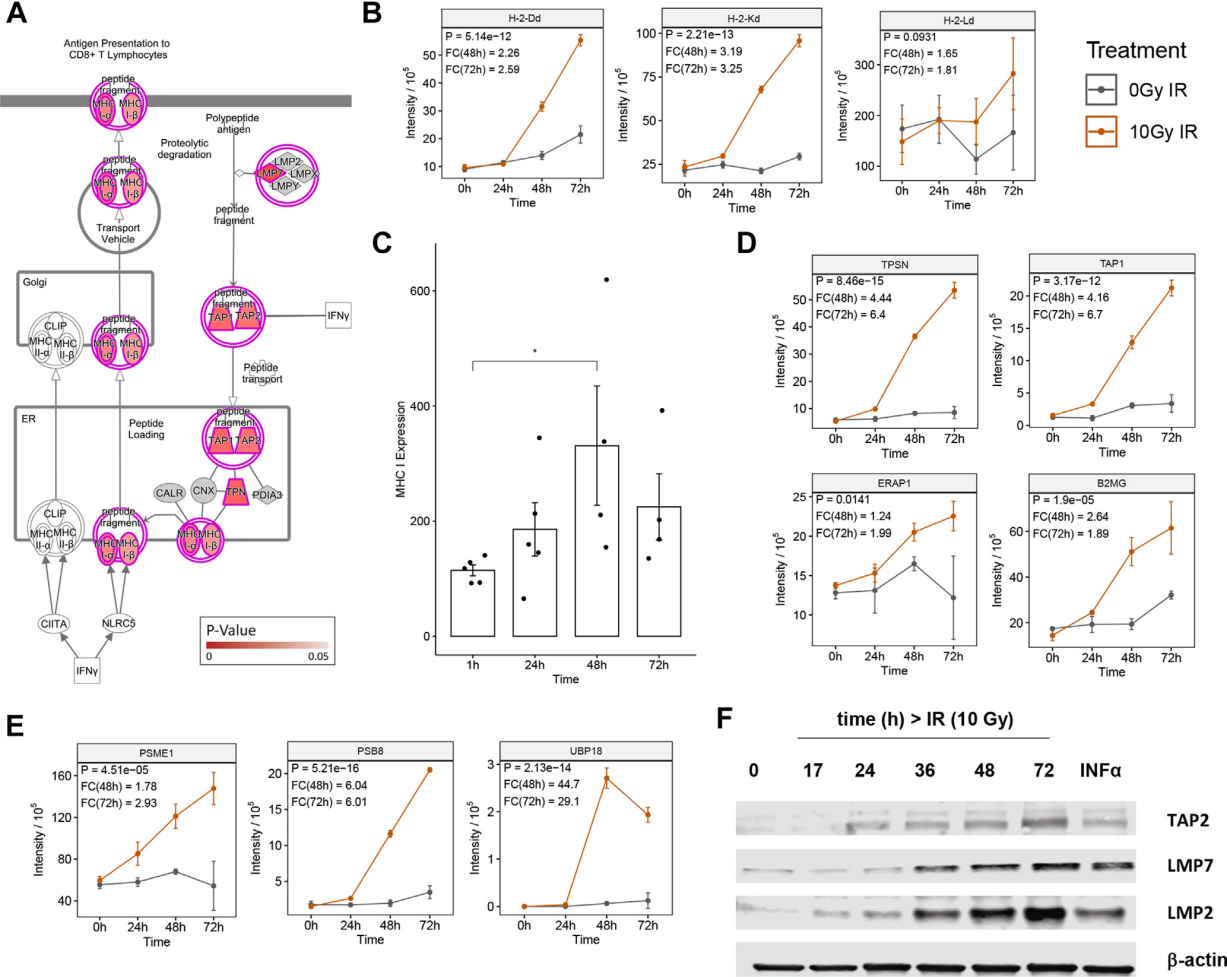
**Analysis of the antigen presentation pathway in the CT26 proteome upon irradiation.***A*, Antigen Presentation Ingenuity Pathway visualized with *p*-values for quantifiable proteins in the CT26 proteome. *B*, intensity plots of proteins encompassing the antigen presentation & ubiquitylation pathways in the CT26 proteome upon irradiation. Points are representative of mean ± SD values of three biological replicates and *p*-values are representative of two-way ANOVA, where *gray* indicates untreated, and *orange* indicates 10 Gy IR treated. *C*, flow cytometric validation of mouse MHC class I expression on the CT26 cell surface upon irradiation. *D*, Western blot validation of mouse immunoproteasome subunits upon irradiation. *p*-values are representative of a paired student’s *t* test and have been denoted by ∗ <0.05. CT, cancer testis.

Among the top 12 canonical pathways the most likely to affect the immunopeptidome were the “Antigen Presentation Pathway” (-logP = 5.67) and the “Protein Ubiquitination Pathway” (-logP = 2.87) (Fig. 2*A*). The antigen presentation pathway has been mapped in Figure 3*A* showing the associated proteins colored by their *p*-value. We confirmed an induction of MHC class I in CT26 in response to radiation: MHC class I molecules present in BALB/c mice showed a significant 2.26-fold and 2.59-fold increase in H-2-Kd (*p* = 5.14e-12) and 3.19-fold and 3.25-fold increase in H-2-Dd (*p* = 2.21e-13) expression levels at the 48 h and 72 h time points respectively. Notably, H-2-Ld (*p* = 0.0931), which is known to be expressed at a much lower level in this cell line, showed a 1.65-fold and 1.81-fold increase at the 48 h and 72 h time points, respectively (14) (Fig. 3*B*). This increase in MHC class I levels was further confirmed in an independent experiment tracking MHC class I surface expression using flow cytometry and mean fluorescent index (Fig. 3*C*). While surface expression of MHC class I in response to radiation was increased significantly after 48 h, MHC class I levels did not change further and had a (nonsignificant) downward trend at 72 h. In contrast, global (intracellular and surface-bound) levels of MHC class I as measured in the proteomics experiment were further increased at 72 h indicating possible changes in intracellular MHC levels at this time posttreatment.

Importantly, an increase in other components of the antigen presentation complex were also observed and include B2M (*p* = 1.9e-05; FC-48 h = 2.64), Tapasin (*p* = 8.46e-15; FC-48 h = 4.44), Tap1 (*p* = 3.17e-12; FC-48 h = 4.16), and ERAP1 (*p* = 0.014; FC-48 h = 1.24) (Fig. 3*D*). In addition, an evaluation of individual proteins involved in the ubiquitin pathway showed an increase in PSME1 (*p* = 4.51e-05; FC-48 h = 1.78), PSMB8 or LMP2 (*p* = 5.21e-16; FC-48 h = 6.04), and UBP18 or USP18 (*p* = 2.13e-14; FC-48 h = 44.7) (Figs. 3*E* and S3). Western blotting for TAP2 and the IFN-inducible immunoproteasome subunits LMP2 and LMP7 also displayed an increase in protein expression after 10 Gy (Fig. 3*F*). Constitutive proteins for all canonical pathways have been included in supplemental Table S1. These data strongly indicate that irradiation has broad effects on peptide processing and therefore a high potential to alter the MHC class I presented antigen landscape of cells.

### Radiation Globally Increases MHC-Associated Peptide Abundance and Breadth and Provides a Subset of Radiation-Specific Peptide Antigens

**Fig 4:**
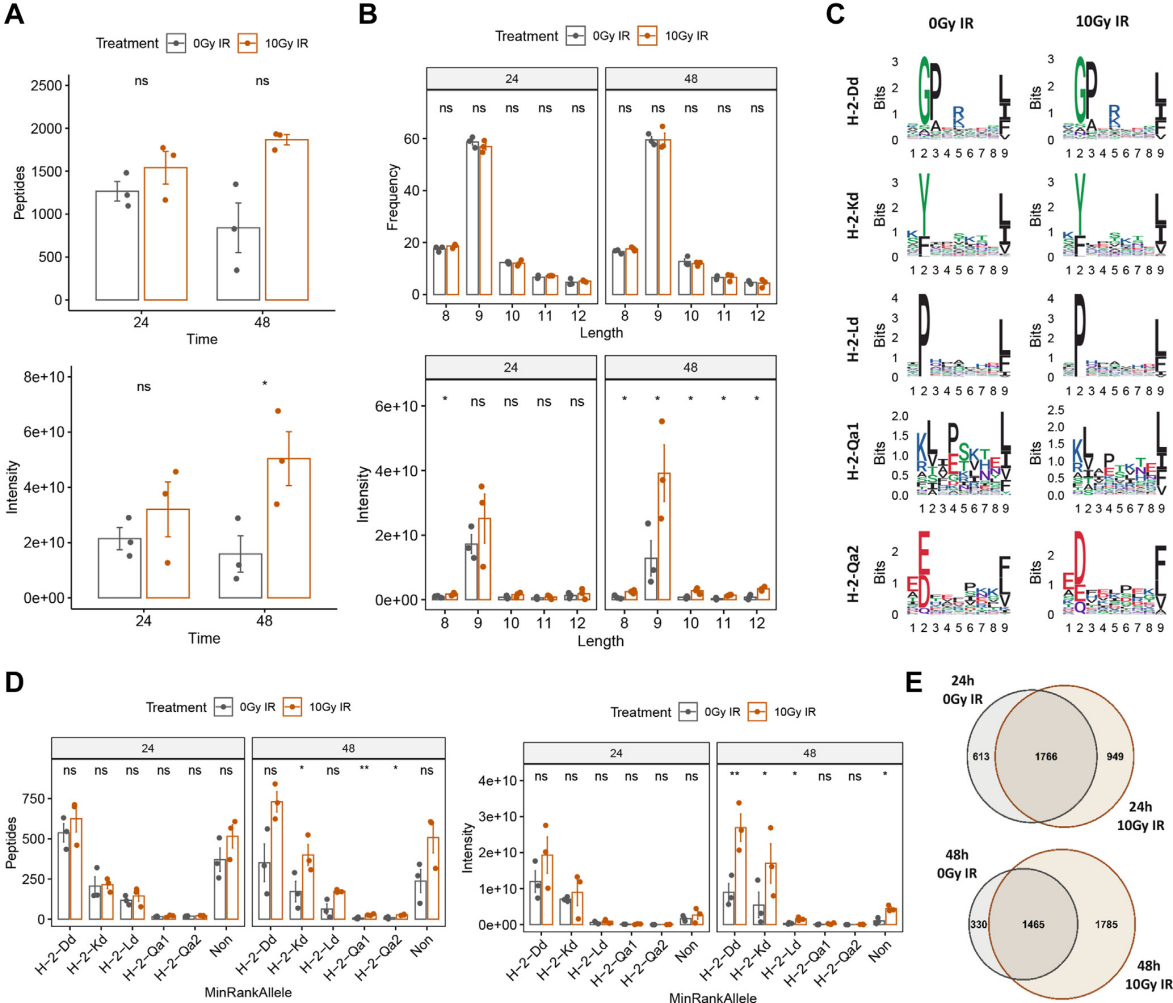
**Analysis of CT26 immunopeptidome upon irradiation.***A*, total MHC peptide intensity at 0 Gy and 10 Gy at 24 and 48 h postirradiation. *B*, length intensity distribution of MHC peptides at 0 Gy and 10 Gy at 24 and 48 h postirradiation. *C*, Seqlogo comparison of MHC-binding motifs before and after irradiation for combined 24 and 48 h time points. Peptides were assessed using NetMHCPan 4.1. *D*, allele-binding intensity distribution at 0 Gy and 10 Gy at 24 and 48 h postirradiation. *E*, unique MHC peptides at 0 Gy and 10 Gy at 24 and 48 h postirradiation. *p*-values are representative of a paired student’s *t* test and have been denoted by ∗ <0.05, ∗∗ <0.01 and ns for not significant. CT, cancer testis.

Due to the prevalence of radiation-independent changes occurring in the proteome at the 72-h time point, only the 24- and 48-h postirradiation time points were selected for the assessment of the immunopeptidome. Firstly, a global quantitative analysis of the immunopeptidome was performed across treatment conditions. Overall, we detected 16,542 peptides across all control and irradiated samples at 24 and 48 h at 1% FDR cut-off with 3913 unique peptides (supplemental Table S2). Our analysis revealed a small increase in the total number of unique peptide sequences at 24 h in comparison to the control (fold-change = 1.21, *p* = 0.33) along with an increase in overall peptide intensity (fold-change = 1.49, *p* = 0.25), while a much greater increase in total unique peptide sequences (fold-change = 2.00, *p* = 0.07) and intensity (fold-change = 3.17, *p* = 0.01) was observed at 48 h postirradiation, in alignment with the proteomics results (Fig. 4*A*). Distribution of the peptides between 8 to 12 amino acids in length showed a preference for 9-mers for the H-2-Kd, H-2-Dd, and H-2-Ld alleles present in BALB/c mice (Fig. 4*B*). Irradiated cells show a similar length distribution of peptide frequency indicating that there are no obvious length-related effects and the overall trend in increased peptide presentation and intensity values upon radiation remain unchanged. Peptides were analyzed using NetMHCpan 4.0 to assess binding to the specific MHC class I alleles H-2-Kd, H-2-Dd, H-2-Ld as well as the nonclassical MHC alleles capable of presenting peptides H-2-Qa1 and H-2-Qa2. Sequence logos of peptides grouped by a binding score of less than 2 were compared for the control and treated conditions and showed no obvious changes in the peptide repertoire (Fig. 4*C*). The distribution of peptides binding to each allele showed that most peptides bind to H-2-Dd followed by H-2-Kd and H-2-Ld (Fig. 4*D*). Allele specific effects of radiation were more prevalent at the 48-h time point. A combined analysis of all three replicates showed an overall increase in unique peptides at both the 24- and 48-h time points with 949 and 1785 unique peptides in the irradiated conditions, respectively (Fig. 4*E*). Venn diagrams showing the overlap between biological replicates are displayed in supplemental Fig. S4. These data support the findings that upregulation of the antigen presentation machinery upon radiation as determined by the proteome analysis results in an increase of MHC-presented peptides in the immunopeptidome. Furthermore, radiation alters the cellular proteome profile, therefore leading to the presence of unique radiation-specific MHC-presented peptides.

### Radiation-Induced Proteomic and Immunopeptidomics Changes are Also Observed in the DNA Damage Repair-Deficient Cell Line MC38

To explore whether these radiation-specific changes observed in CT26 were cell-line specific, a series of cross-validation experiments were performed in second colorectal cell line, MC38, which is derived from the C57BL6 mouse strain which carries the H-2-Kb and H-2-Db alleles. The MC38 cell line contains characteristics which include the mutational signature for DNA mismatch-repair deficiency and is also responsive to immune-checkpoint inhibition indicating its validity to be used to model microsatellite-instable colorectal cancer (27, 28). DNA-damage repair deficiency is a discerning factor, which can stratify colorectal cancer patient outcomes during radiotherapy (29).

First, a label-free quantitative proteomics experiment was performed to assess the differential expression of 3262 quantifiable proteins at 24 h and 48 h post 10 Gy irradiation. Differentially expressed proteins were significantly increased after irradiation in a time-dependent manner (time postirradiation, downregulated proteins, upregulated proteins; 24 h, 22↓, 16↑; 48 h, 6↓, 65↑) (supplemental Fig. S5*A*). An Ingenuity pathway analysis at the 48-h time point (*p* > 0.05, FC > 1.5) showed similar pathways to CT26 with Interferon Signaling (-logP = 8.79), Activation of IRF by Cytosolic Pattern Recognition Receptors (-logP = 8.02), and the Antigen Presentation Pathway (-logP = 6.2) being the top three pathways (supplemental Fig. S5*B*). A selection of individual proteins from the antigen presentation pathway were extracted to demonstrate the changes between the 24 h and 48 h time points including H-2-Kb (FC-24 h = 1.35, FC-48 h = 2.71), H-2-Db (FC-24 h = 1.32, FC-48 h = 2.11), B2MG (FC-24 h = 1.64, FC-48 h = 2.96), TAP1 (FC-24 h = 1.18, FC-48 h = 3.32), TAP2 (FC-24 h = 1.26, FC-48 h = 2.62), TPSN (FC-24 h = 1.3, FC-48 h = 2.74), PSB8 (FC-24 h = 1.28, FC-48 h = 1.28), PSME1 (FC-24 h = 1.05, FC-48 h = 1.23), and ERAP1 (FC-24 h = 1.14, FC-48 h = 1.35) (supplemental Fig. S5*C*). Again, the encompassing proteins of the “Antigen Presentation Pathway” increase upon radiation but slightly less so compared to the changes observed in CT26.

We then tested whether we could also observe an increase in peptide presentation by MHC class I. MC38 cells were treated with 10 Gy Irradiation at the 24-h time point, and an immunopeptidomics experiment was performed to detect a total of 15,915 peptides from the control and irradiated samples at the 1% FDR cut-off which met the criteria of 8 to 14-mers with a netMHCpan-binding score <2 which constituted 4017 unique peptides. A slight but significant increase in the total number of peptides was observed upon treatment (fold-change = 1.13, *p* = 0.037) (supplemental Fig. S5*D*). Distribution of peptides between 8 to 12 amino acids showed a preference for both 8-mers and 9-mers, as expected from the H-2-b haplotype (supplemental Fig. S5*E*). This trend was observed across both H-2-Db and H-2-Kb but with statistical significance only observed in the latter (supplemental Fig. S5*F*). A combined analysis of three replicates showed an overall increase in unique peptides totaling 429 unique peptides in the irradiated condition (supplemental Fig. S5*G*). A comparison across both CT26 and the MC38 datasets showed an overlap of 92 source proteins among radiation-specific peptides (supplemental Fig. S5*H*). Furthermore, an overrepresentation analysis using the ingenuity pathways analysis tool grouped several common source proteins from pathways which were upregulated in radiation including the “Protein Ubiquitination Pathway” and the “Antigen Presentation Pathway” (supplemental Fig. S5*I*). These findings demonstrate that radiation-specific increases in the antigen presentation pathway in mouse colorectal cancer cell lines can be observed across mouse strains carrying differing MHC haplotypes and irrespective of microsatellite instability; however, the extent to which these changes are observed vary.

### Radiation-Induced Upregulation and Downregulation of the Proteome Does Not Directly Translate Into Changes in Antigen Presentation

**Fig 5:**
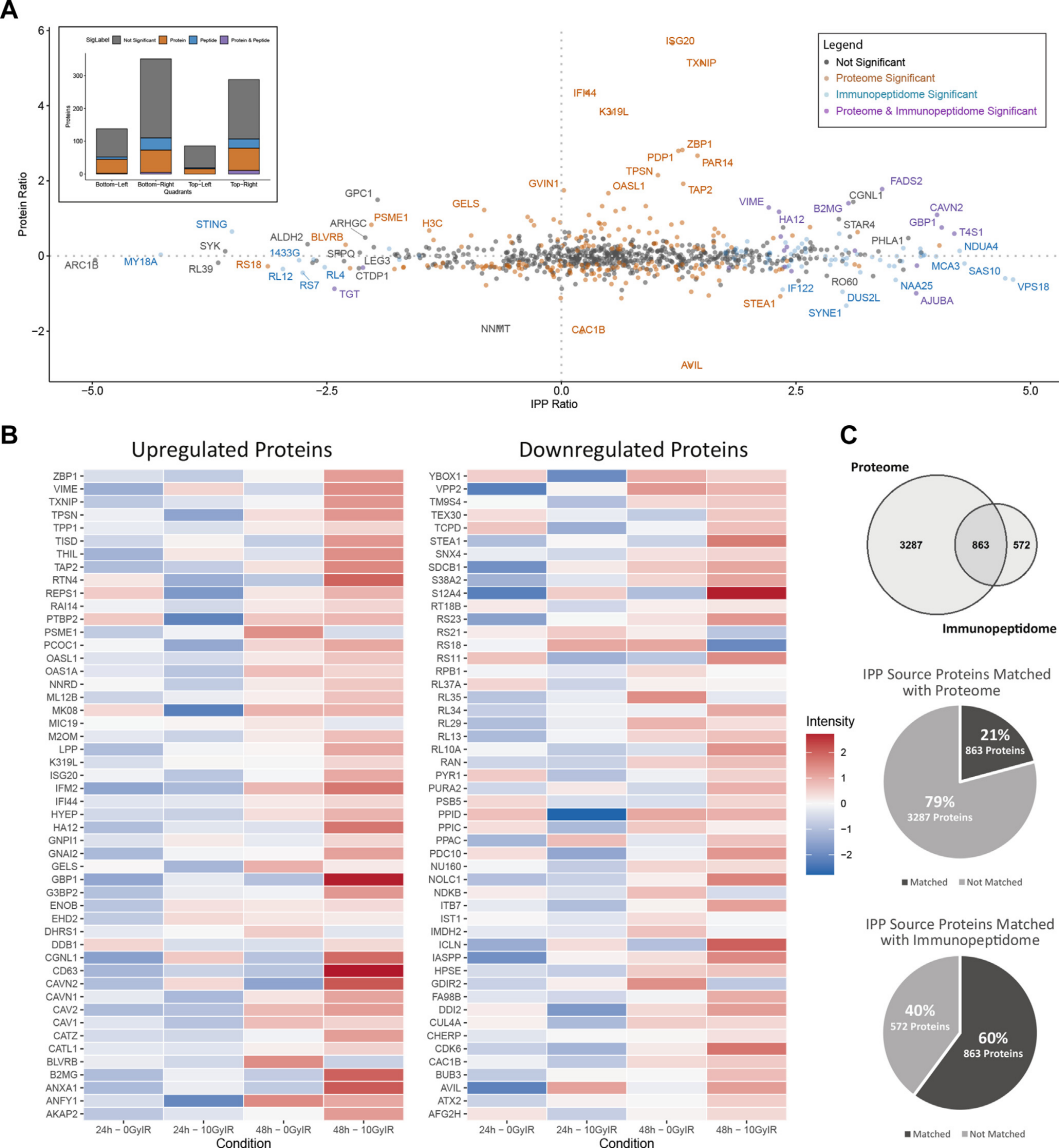
**Cross analysis of CT26 proteome and immunopeptidome and radiation-induced antigens.***A*, correlation plot of the proteome protein and the immunopeptidome peptide ratio at 48 h post 10 Gy. Significantly differentially expressed proteins and peptides are indicated by their colors. A bar chart to quantify proteins in each quadrant is detailed to show an alternative representation of the data. *B*, centered heatmap of changes in the immunopeptidome in significantly upregulated (*left*) and downregulated (*right*) proteins in the proteome. *C*, coverage of MHC peptide source proteins in the proteome (*top*). Coverage of proteome among source proteins in the immunopeptidome (*middle*). Overlap of proteome and immunopeptidome source proteins (*bottom*). CT, cancer testis.

MHC class I peptides originate from the proteasomal cleavage of endogenous proteins; allele-dependent MHC-binding motifs are one factor which drives the number of peptides which may originate from each protein source, however, other factors including protein expression may contribute to this relationship. Therefore, the next objective was to understand whether changes in the proteome were driving the changes observed in the immunopeptidome. To do this, a quantitative analysis of all peptides in the immunopeptidomics data was performed and peptides were summed based on their source proteins using the progenesis software algorithm. Quantification was performed using all peptides which could originate from the relevant source protein. The protein ratio from the proteomics data was plotted against the source protein ratio from the immunopeptidomics data (IPP Ratio) at the 48-h time point. A *p* value significance threshold of <0.05 was used to indicate differentially expressed proteins from the proteome analysis (orange), source proteins from the immunopeptidome analysis (blue), and proteins where both proteomic and immunopeptidomics analyses were significant (purple) (Fig. 5*A*). This indicated statistical significance for each dataset and their overlap. Interestingly, several proteins which were significantly increased in the proteome were not as significantly increased in the immunopeptidome including TPSN and ISG20. Similarly, many source proteins which were significantly increased in the immunopeptidome did not correspond to changes in the proteome. Notably, both the downregulation and upregulation of proteins in the proteome can result in the presentation of MHC peptides and vice versa.

However, several significant proteins overlapped the immunopeptidome and the proteome. While the main trend observed in the analysis reveals a linear relationship between protein expression and peptide presentation, some peptides display evidence of active degradation. For example, proteins in the top right quadrant of Figure 5*A* represent those proteins which are being presented more abundantly on MHC class I and have upregulated protein expression, including H-2-Kd and B2M, which directly facilitate antigen presentation. The top left quadrant of Figure 5*A* shows no significantly correlated proteins, indicating that most upregulated proteins do not tend to show a decrease in their MHC class I presentation. The proteins in the bottom left quadrant of Figure 5*A* show proteins which are downregulated and reduced on MHC class I, including arginine-tRNA ligase which is linked with protein synthesis. Finally, the bottom right quadrant of Figure 5*A* shows proteins which are downregulated in the proteome but upregulated in the immunopeptidome, indicating presentation of degraded proteins. Among several ribosomal proteins, this group also includes the LIM domain-containing protein AJUBA which is involved in several pathways including IL-1 signaling (30).

This analysis demonstrates that both proteins that are upregulated or downregulated due to radiation treatment can have an increased peptide presentation by MHC class I. This relationship is further highlighted in Figure 5*B* which shows changes in the immunopeptidome for the most significantly upregulated and downregulated proteins in the proteome. Again, a global increase in MHC class I peptide presentation is observed at the individual peptide level largely at the 48-h time point, correlating closely with the results displayed in Figure 4.

While the correlation between protein expression and their associated antigens provides a quantitative understanding of how antigen processing is altered in the context of radiation, there were only 863 proteins that overlapped the proteome and immunopeptidome (Fig. 5*C*). Furthermore, this only included proteins and peptides which were deemed quantifiable, being represented across conditions. An additional 572 unique peptides in the immunopeptidome originated from proteins which were not detected in the proteome.

### Radiation Induces Radiation-Specific Peptide Antigens Including Peptides Associated with Catecholamine Signaling

Our final objective was to investigate the antigens found only in the context of radiation, including the 572 peptides which did not overlap with the proteome. To do this, the dataset was expanded to include additional CT26 control immunopeptidomics datasets which had been previously generated under the same conditions. We first ranked all MHC peptides by their binding score for the minimum ranking binding allele. Only peptides below a threshold of 0.5 rank binding score, which are representative of strong binders were included in the dataset (Table 1). Source proteins where multiple unique MHC class I peptides appeared were hypothesized as being more closely linked with radiation. Accordingly, two proteins had multiple associated peptides including ADCY7 and S14L1. Source proteins which were unique to either of the radiation-treated datasets and that were not present in any of the control samples underwent a qualitative assessment for overrepresented pathways. The top two pathways were associated with Dopamine Receptor Signaling (-log *p* = 5.14) and Serotonin Receptor Signaling (-log *p* = 4.88). The full canonical pathway analysis has been included in supplemental Fig. S6 and constitutive proteins for all canonical pathways have been included in supplemental Table S3. All annotations have been indicated in Table 1. Several peptides and proteins which match to known CT (cancer testis) antigens were also identified; however, they were not regulated in a radiation-dependent manner (supplemental Fig. S8).

**Table 1:** Radiation-specific peptides presented on the CT26 immunopeptidome

Peptide	Allele	Rank	Entry	Protein name
AYSSLVTSL	H-2-Kd	0.0029	PLIN4_MOUSE	Perilipin-4 (Adipocyte protein S3-12)
RGPLHHATI	H-2-Dd	0.0033	ACAP1_MOUSE	Arf-GAP with coiled-coil, ANK repeat, and PH domain-containing protein 1 (Centaurin-beta-1) (Cnt-b1)
SGPDRTVQF	H-2-Dd	0.0038	I12R1_MOUSE	Interleukin-12 receptor subunit beta-1 (IL-12 receptor subunit beta-1) (IL-12R subunit beta-1) (IL-12R-beta-1) (IL-12 receptor beta component) (CD antigen CD212)
HYLDTTTLI	H-2-Kd	0.0042	CRKL_MOUSE	Crk-like protein
SYLGNDTRI	H-2-Kd	0.0043	AGRA2_MOUSE	Adhesion G protein–coupled receptor A2 (G protein–coupled receptor 124) (Tumor endothelial marker 5)
YYQGVIQQI	H-2-Kd	0.0051	KATL1_MOUSE	Katanin p60 ATPase-containing subunit A-like 1 (Katanin p60 subunit A-like 1) (EC 5.6.1.1) (p60 katanin-like 1)
KYLTSVVKL	H-2-Kd	0.0056	CY24A_MOUSE	Cytochrome b-245 light chain (Cytochrome b(558) alpha chain) (Cytochrome b558 subunit alpha) (Neutrophil cytochrome b 22 kDa polypeptide) (Superoxide-generating NADPH oxidase light chain subunit) (p22 phagocyte B-cytochrome) (p22-phox) (p22phox)
RGPLHHATV	H-2-Dd	0.0058	ACAP2_MOUSE	Arf-GAP with coiled-coil, ANK repeat, and PH domain-containing protein 2 (Centaurin-beta-2) (Cnt-b2)
SGPERAAFI	H-2-Dd	0.0061	HAP1_MOUSE	Huntingtin-associated protein 1 (HAP-1)
SYSGSIQSL	H-2-Kd	0.0062	RHG42_MOUSE	Rho GTPase-activating protein 42 (Rho-type GTPase-activating protein 42)
VPLASKYNL	H-2-Ld	0.0067	INCE_MOUSE	Inner centromere protein
SPLKAINSF	H-2-Ld	0.0069	BIR1E_MOUSE	Baculoviral IAP repeat-containing protein 1e (Neuronal apoptosis inhibitory protein 5)
AGPLKGVQL	H-2-Dd	0.007	LTK_MOUSE	Leukocyte tyrosine kinase receptor (EC 2.7.10.1)
KGPLINSEF	H-2-Dd	0.0071	BGAL_MOUSE	Beta-galactosidase (EC 3.2.1.23) (Acid beta-galactosidase) (Lactase)
SYAVGLAAL	H-2-Kd	0.0071	TM160_MOUSE	Transmembrane protein 160
SGPTIQDYL	H-2-Dd	0.008	F133B_MOUSE	Protein FAM133B
TGPPVSELI	H-2-Dd	0.0082	NR2F6_MOUSE	Nuclear receptor subfamily 2 group F member 6 (COUP transcription factor 3) (COUP-TF3) (V-erbA-related protein 2) (EAR-2)
IGPGPVELI	H-2-Dd	0.0082	PTH2_MOUSE	Peptidyl-tRNA hydrolase 2, mitochondrial (PTH 2) (EC 3.1.1.29)
TYHEVVDEI	H-2-Kd	0.0087	PR38B_MOUSE	Pre-mRNA-splicing factor 38B
VGPSVPSVI	H-2-Dd	0.0087	SELN_MOUSE	Selenoprotein N (SelN)
IPQLSSHTL	H-2-Ld	0.0088	EFMT1_MOUSE	EEF1A lysine methyltransferase 1 (EC 2.1.1.-) (N(6)-adenine-specific DNA methyltransferase 2) (Protein-lysine N-methyltransferase N6amt2)
SPQKHGVLL	H-2-Ld	0.0089	SWET1_MOUSE	Sugar transporter SWEET1 (MmSWEET1) (RAG1-activating protein 1) (Solute carrier family 50 member 1)
IGPNAGLGF	H-2-Dd	0.0092	VMAT1_MOUSE	Chromaffin granule amine transporter (Solute carrier family 18 member 1) (Vesicular amine transporter 1) (VAT1)
LPFQGKVNL	H-2-Ld	0.0094	TRM11_MOUSE	tRNA (guanine (10)-N2)-methyltransferase homolog (EC 2.1.1.-) (tRNA guanosine-2′-O-methyltransferase TRM11 homolog)
IGPDVTDIL	H-2-Dd	0.0094	MED1_MOUSE	Mediator of RNA polymerase II transcription subunit 1 (Mediator complex subunit 1) (Peroxisome proliferator-activated receptor-binding protein) (PBP) (PPAR-binding protein) (Thyroid hormone receptor-associated protein complex 220 kDa component) (Trap220) (Thyroid receptor-interacting protein 2) (TR-interacting protein 2) (TRIP-2)
LGPQAGRTL	H-2-Dd	0.0096	CMIP_MOUSE	C-Maf-inducing protein (c-Mip)
RGPQGYGFNL	H-2-Dd	0.0097	NHRF2_MOUSE	Na(+)/H(+) exchange regulatory cofactor NHE-RF2 (NHERF-2) (NHE3 kinase A regulatory protein E3KARP) (Octs2) (SRY-interacting protein 1) (SIP-1) (Sodium-hydrogen exchanger regulatory factor 2) (Solute carrier family 9 isoform A3 regulatory factor 2) (Tyrosine kinase activator protein 1) (TKA-1)
VPSENVLNF	H-2-Ld	0.0099	SPAG5_MOUSE	Sperm-associated antigen 5 (Mastrin) (Mitotic spindle-associated protein p126) (MAP126)
YYNAQNTSV	H-2-Kd	0.01	AKAP8_MOUSE	A-kinase anchor protein 8 (AKAP-8) (A-kinase anchor protein 95 kDa) (AKAP 95)
SGPDRDAIL^c^	H-2-Dd	0.0108	TTK_MOUSE	Dual specificity protein kinase TTK (EC 2.7.12.1) (ESK) (PYT)
VGPPALSRV	H-2-Dd	0.011	COG1_MOUSE	Conserved oligomeric Golgi complex subunit 1 (COG complex subunit 1) (Component of oligomeric Golgi complex 1) (Low density lipoprotein receptor defect B-complementing protein)
IYNQVKQII	H-2-Kd	0.0112	DLG1_MOUSE	Disks large homolog 1 (Embryo-dlg/synapse-associated protein 97) (E-dlg/SAP97) (Synapse-associated protein 97) (SAP-97) (SAP97)
IGPSQGNGF	H-2-Dd	0.0113	RECQ5_MOUSE	ATP-dependent DNA helicase Q5 (EC 3.6.4.12) (DNA helicase, RecQ-like type 5) (RecQ5) (RECQL5beta) (RecQ protein-like 5)
EYFSSTSEL^a^	H-2-Kd	0.0115	DAPK3_MOUSE	Death-associated protein kinase 3 (DAP kinase 3) (EC 2.7.11.1) (DAP-like kinase) (Dlk) (MYPT1 kinase) (ZIP-kinase)
VYFVQKNSL^b^	H-2-Kd	0.0152	S14L1_MOUSE	SEC14-like protein 1
RPQVAKTLL	H-2-Ld	0.0161	HECD1_MOUSE	E3 ubiquitin-protein ligase HECTD1 (EC 2.3.2.26) (HECT domain-containing protein 1) (HECT-type E3 ubiquitin transferase HECTD1) (Protein open mind)
APHKTGLEL	H-2-Ld	0.0163	HJURP_MOUSE	Holliday junction recognition protein (Fetal liver expressing gene 1 protein homolog) (mFleg1)
FYGATGTLL	H-2-Kd	0.0168	FADS3_MOUSE	Fatty acid desaturase 3 (EC 1.14.19.-) (Delta (13) fatty acid desaturase) (Delta (13) desaturase)
AGPSAFNI	H-2-Dd	0.0186	WAC_MOUSE	WW domain-containing adapter protein with coiled-coil
APARAILSL	H-2-Ld	0.0189	S23A2_MOUSE	Solute carrier family 23 member 2 (Na(+)/L-ascorbic acid transporter 2) (Sodium-dependent vitamin C transporter 2) (SVCT-2) (mSVCT2) (Yolk sac permease-like molecule 2)
VPQQILQGL	H-2-Ld	0.0195	ABHD6_MOUSE	Monoacylglycerol lipase ABHD6 (EC 3.1.1.23) (2-arachidonoylglycerol hydrolase) (Abhydrolase domain-containing protein 6)
SPSPAILGL	H-2-Ld	0.0197	FGD3_MOUSE	FYVE, RhoGEF, and PH domain-containing protein 3
VYKASLNLI	H-2-Kd	0.0197	IMA1_MOUSE	Importin subunit alpha-1 (Importin alpha P1) (Karyopherin subunit alpha-2) (Pendulin) (Pore targeting complex 58 kDa subunit) (PTAC58) (RAG cohort protein 1) (SRP1-alpha)
TGPATISL	H-2-Dd	0.0198	TMUB2_MOUSE	Transmembrane and ubiquitin-like domain-containing protein 2
IPQQLVERL	H-2-Ld	0.0204	UBP7_MOUSE	Ubiquitin carboxyl-terminal hydrolase 7 (EC 3.4.19.12) (Deubiquitinating enzyme 7) (Herpesvirus-associated ubiquitin-specific protease) (mHAUSP) (Ubiquitin thioesterase 7) (Ubiquitin-specific–processing protease 7)
LGPLAGDNF	H-2-Dd	0.0216	OSGI1_MOUSE	Oxidative stress-induced growth inhibitor 1
RPHSVRDLF	H-2-Ld	0.022	REV1_MOUSE	DNA repair protein REV1 (EC 2.7.7.-) (Rev1-like terminal deoxycytidyl transferase)
TGPNNSNTTF	H-2-Dd	0.023	BCLF1_MOUSE	Bcl-2-associated transcription factor 1 (Btf)
TYTRGLTGL	H-2-Kd	0.0241	HYAL2_MOUSE	Hyaluronidase-2 (Hyal-2) (EC 3.2.1.35) (Hyaluronoglucosaminidase-2)
VGPRRGDFTRL	H-2-Dd	0.0253	CAMP3_MOUSE	Calmodulin-regulated spectrin-associated protein 3 (Marshalin) (Protein Nezha)
IGAARGLLL	H-2-Dd	0.0253	AGRIN_MOUSE	Agrin [Cleaved into: Agrin N-terminal 110 kDa subunit; Agrin C-terminal 110 kDa subunit; Agrin C-terminal 90 kDa fragment (C90); Agrin C-terminal 22 kDa fragment (C22)]
IGPYYRKL	H-2-Dd	0.0261	TMCO3_MOUSE	Transmembrane and coiled-coil domain-containing protein 3
SFLETVNQL	H-2-Kd	0.0264	TWSG1_MOUSE	Twisted gastrulation protein homolog 1
SYGYPPSSL	H-2-Kd	0.029	YTHD3_MOUSE	YTH domain-containing family protein 3
KPEQFLHEL	H-2-Ld	0.0298	FMN1_MOUSE	Formin-1 (Limb deformity protein)
LGPVISTGL	H-2-Dd	0.03	BAP1_MOUSE	Ubiquitin carboxyl-terminal hydrolase BAP1 (EC 3.4.19.12) (BRCA1-associated protein 1) (Ubiquitin C-terminal hydrolase X4) (UCH-X4)
HPLLNVHDL	H-2-Ld	0.0332	PDPR_MOUSE	Pyruvate dehydrogenase phosphatase regulatory subunit, mitochondrial (PDPr)
KGFEREYRL^a^^b^	H-2-Dd	0.0332	ADCY7_MOUSE	Adenylate cyclase type 7 (EC 4.6.1.1) (ATP pyrophosphate-lyase 7) (Adenylate cyclase type VII) (Adenylyl cyclase 7)
SYKRQNEAI	H-2-Kd	0.0342	PPHLN_MOUSE	Periphilin-1
KPQKFINDL	H-2-Ld	0.0351	BEND6_MOUSE	BEN domain-containing protein 6
RGPVVPKPQL	H-2-Dd	0.0359	SBP2L_MOUSE	Selenocysteine insertion sequence-binding protein 2-like (SECIS-binding protein 2-like)
NYARPKQFI	H-2-Kd	0.0362	PALLD_MOUSE	Palladin
KGAPHEILI^b^	H-2-Dd	0.0397	S14L1_MOUSE	SEC14-like protein 1
ASIPVNLRL	H-2-Qa1	0.0427	AMPE_MOUSE	Glutamyl aminopeptidase (EAP) (EC 3.4.11.7) (Aminopeptidase A) (AP-A) (BP-1/6C3 antigen) (CD antigen CD249)
KGPDHGVLDAL	H-2-Dd	0.0435	ZSWM9_MOUSE	Uncharacterized protein ZSWIM9
EDFDSKLSF	H-2-Qa2	0.0461	EFHD2_MOUSE	EF-hand domain-containing protein D2 (Swiprosin-1)
YYGGVEHEI	H-2-Kd	0.0476	SGSM2_MOUSE	Small G protein signaling modulator 2 (RUN and TBC1 domain-containing protein 1)
KPHSGFHVAF	H-2-Ld	0.0538	MICU2_MOUSE	Calcium uptake protein 2, mitochondrial (EF-hand domain-containing family member A1)
LPSPAGPIL	H-2-Ld	0.0572	AFF4_MOUSE	AF4/FMR2 family member 4
SFSESISAL	H-2-Kd	0.071	SYBU_MOUSE	Syntabulin (Golgi-localized syntaphilin-related protein) (m-Golsyn) (Syntaxin-1-binding protein)
LPYNHQHEYF	H-2-Ld	0.0723	FADS2_MOUSE	Acyl-CoA 6-desaturase (EC 1.14.19.3) (Delta (6) fatty acid desaturase) (D6D) (Delta (6) desaturase) (Delta-6 desaturase) (Fatty acid desaturase 2)
LGPKVEAL	H-2-Dd	0.076	HDGR2_MOUSE	Hepatoma-derived growth factor–related protein 2 (HRP-2)
ADHLITENF	H-2-Qa2	0.0767	VP26C_MOUSE	Vacuolar protein sorting-associated protein 26C (Down syndrome critical region protein 3 homolog) (Down syndrome critical region protein A homolog)
KGPISEEGL	H-2-Dd	0.0774	PEAR1_MOUSE	Platelet endothelial aggregation receptor 1 (mPEAR1) (Jagged and Delta protein) (Protein Jedi) (Multiple epidermal growth factor-like domains protein 12) (Multiple EGF-like domains protein 12)
TGPQARTI	H-2-Dd	0.0792	ITSN1_MOUSE	Intersectin-1 (EH and SH3 domains protein 1)
LGPPVQQI	H-2-Dd	0.0814	USO1_MOUSE	General vesicular transport factor p115 (Protein USO1 homolog) (Transcytosis-associated protein) (TAP) (Vesicle-docking protein)
HPGQHLIGL	H-2-Ld	0.0864	VEZA_MOUSE	Vezatin
SAPTLEDHF	H-2-Dd	0.0951	MFA1B_MOUSE	Microfibrillar-associated protein 1B (Spliceosome B complex protein MFAP1B)
NDSVIVDTF	H-2-Qa2	0.0981	UBP11_MOUSE	Ubiquitin carboxyl-terminal hydrolase 11 (EC 3.4.19.12) (Deubiquitinating enzyme 11) (Ubiquitin thioesterase 11) (Ubiquitin-specific-processing protease 11)
NGPNHGKAF	H-2-Dd	0.1017	ERI2_MOUSE	ERI1 exoribonuclease 2 (EC 3.1.-.-) (Exonuclease domain-containing protein 1)
FYEKVQSDL	H-2-Kd	0.1079	S38A1_MOUSE	Sodium-coupled neutral amino acid transporter 1 (Amino acid transporter A1) (MNat2) (N-system amino acid transporter 2) (Solute carrier family 38 member 1) (System A amino acid transporter 1) (System N amino acid transporter 1)
NYKLLKTGI	H-2-Kd	0.1134	STAG2_MOUSE	Cohesin subunit SA-2 (SCC3 homolog 2) (Stromal antigen 2)
KFDTVKSVL	H-2-Kd	0.1239	COP1_MOUSE	E3 ubiquitin-protein ligase COP1 (EC 2.3.2.27) (Constitutive photomorphogenesis protein 1 homolog) (mCOP1) (RING finger and WD repeat domain protein 2) (RING-type E3 ubiquitin transferase RFWD2)
VGPTQNRI^a^	H-2-Dd	0.1291	AOFB_MOUSE	Amine oxidase [flavin-containing] B (EC 1.4.3.4) (Monoamine oxidase type B) (MAO-B)
NGPTHSSTLF	H-2-Dd	0.1405	TE2IP_MOUSE	Telomeric repeat-binding factor 2-interacting protein 1 (TERF2-interacting telomeric protein 1) (TRF2-interacting telomeric protein 1) (Repressor/activator protein 1 homolog) (RAP1 homolog)
TFVVSRTEV	H-2-Kd	0.1463	CNNM2_MOUSE	Metal transporter CNNM2 (Ancient conserved domain-containing protein 2) (mACDP2) (Cyclin-M2)
KPYNKIVSHLL	H-2-Ld	0.1607	ERR3_MOUSE	Estrogen-related receptor gamma (Estrogen receptor-related protein 3) (Nuclear receptor subfamily 3 group B member 3)
TGAFHKHQL^a^^b^	H-2-Dd	0.1612	ADCY7_MOUSE	Adenylate cyclase type 7 (EC 4.6.1.1) (ATP pyrophosphate-lyase 7) (Adenylate cyclase type VII) (Adenylyl cyclase 7)
EPFRLEHNL	H-2-Ld	0.1656	ZMIZ1_MOUSE	Zinc finger MIZ domain-containing protein 1 (PIAS-like protein Zimp10) (Retinoic acid–induced protein 17)
EDLHLGTSF	H-2-Qa2	0.1936	TLS1_MOUSE	Telomere length and silencing protein 1 homolog
VGPKRKEEAI	H-2-Dd	0.2178	TSYL2_MOUSE	Testis-specific Y-encoded–like protein 2 (TSPY-like protein 2) (CASK-interacting nucleosome assembly protein) (Differentially expressed nucleolar TGF-beta1 target protein)
QGPDITLSKL	H-2-Dd	0.2519	APC5_MOUSE	Anaphase-promoting complex subunit 5 (APC5) (Cyclosome subunit 5)
KLQQALTQL	H-2-Qa1	0.2707	AMOL1_MOUSE	Angiomotin-like protein 1 (junction-enriched and junction-associated protein) (JEAP)
TGPLQHGI	H-2-Dd	0.2911	5NTC_MOUSE	Cytosolic purine 5′-nucleotidase (EC 3.1.3.5) (Cytosolic 5′-nucleotidase II)
AAPRSEEL	H-2-Dd	0.2912	IRX2_MOUSE	Iroquois-class homeodomain protein IRX-2 (Homeodomain protein IRXA2) (Iroquois homeobox protein 2) (Iroquois-class homeobox protein Irx6)
GGPSRGPLDGF	H-2-Dd	0.3029	EMIL1_MOUSE	EMILIN-1 (Elastin microfibril interface-located protein 1) (Elastin microfibril interfacer 1)
VGAVRLLSV	H-2-Dd	0.3097	GTR6_MOUSE	Solute carrier family 2, facilitated glucose transporter member 6 (Glucose transporter type 6) (GLUT-6)
LGPFRTGSNL	H-2-Dd	0.3282	FNIP2_MOUSE	Folliculin-interacting protein 2 (O6-methylguanine–induced apoptosis 1 protein)
AGIIHKDLI	H-2-Dd	0.3389	ASH1L_MOUSE	Histone-lysine N-methyltransferase ASH1L (EC 2.1.1.359) (EC 2.1.1.367) (ASH1-like protein) (Absent small and homeotic disks protein 1 homolog)
KLVEGRTHI	H-2-Kd	0.3568	CO4B_MOUSE	Complement C4-B [Cleaved into: Complement C4 beta chain; Complement C4 alpha chain; C4a anaphylatoxin; Complement C4 gamma chain]
GIQPSPVLL	H-2-Qa1	0.3576	NB5R1_MOUSE	NADH-cytochrome b5 reductase 1 (b5R.1) (EC 1.6.2.2) (NAD(P)H:quinone oxidoreductase type 3 polypeptide A2)
IPSQGPHPDL	H-2-Ld	0.3666	NRADD_MOUSE	Death domain-containing membrane protein NRADD (Neurotrophin receptor homolog-2) (NRH2) (Neurotrophin receptor-alike death domain protein)
AQPGRSSSL	H-2-Dd	0.3795	RHG20_MOUSE	Rho GTPase-activating protein 20 (RA and RhoGAP domain-containing protein) (RARhoGAP) (Rho-type GTPase-activating protein 20)
APLGASPRLVL	H-2-Ld	0.3838	PMVK_MOUSE	Phosphomevalonate kinase (PMKase) (EC 2.7.4.2)
SYKPVRSV	H-2-Kd	0.3856	ERGI2_MOUSE	Endoplasmic reticulum-Golgi intermediate compartment protein 2
YSFGRTTI	H-2-Dd	0.4459	CSCL1_MOUSE	CSC1-like protein 1 (Transmembrane protein 63A)
EAFEHENKF	H-2-Ld	0.4838	VPS29_MOUSE	Vacuolar protein sorting-associated protein 29 (Vesicle protein sorting 29)
FEADPERFNNF	H-2-Qa2	0.4965	G6PI_MOUSE	Glucose-6-phosphate isomerase (GPI) (EC 5.3.1.9) (Autocrine motility factor) (AMF) (Neuroleukin) (NLK) (Phosphoglucose isomerase) (PGI) (Phosphohexose isomerase) (PHI)

Noteworthy peptides have been highlighted in *gray*.

a Peptides involved with catecholamine receptor signaling ingenuity pathway.

b Source proteins with multiple representative MHC peptides.

c CT antigens.

### Radiation Does Not Alter Global Posttranslational Modifications on the CT26 Immunopeptidome

While radiation is known to induce certain modifications in the proteome, its effect on posttranslational modifications (PTMs) in the immunopeptidome are unknown (31). We first determined whether the inclusion of a PTM search criteria would alter the overall findings observed in the standard analysis. To do this, two different search methods were used including firstly, PEAKS PTM which retrospectively analyses data for 313 possible in-built modifications (supplemental Fig. S9*A*) and secondly, the inclusion of common variable modifications in the initial search criteria (supplemental Fig. S9*B*). The radiation-specific increase in total peptides and their relative intensities were still prevalent after performing both methods; however, a greater number of overall identifications were observed using the PEAKS PTM method. Furthermore, a separate analysis of only the modified and unmodified peptides verified that the radiation-specific induction of peptide presentation was independent of modification. We chose to use PEAKS PTM for our downstream analysis as it was deemed a more unbiased method to assess PTMs on MHC peptides, where the influence of radiation is currently unclear.

To assess whether the potential for modification was increased upon treatment, PTM data was represented as a ratio of posttranslationally modified peptides to unmodified peptides within each treatment condition (supplemental Fig. S10). The total PTMs among the entire immunopeptidome remained unchanged between treatment conditions (supplemental Fig. S10*A*); however, upon further analysis of PTMs on each position of 9-mers, modifications were significantly increased on position 7 in irradiated cells at both 24 and 48-h time points (supplemental Fig. S10*B*). An analysis of individual PTMs showed no significant differences upon radiation treatment (supplemental Fig. S10*C*).

### Radiation Increases Presentation of the MTCH1 Neoantigen in CT26

**Fig 6:**
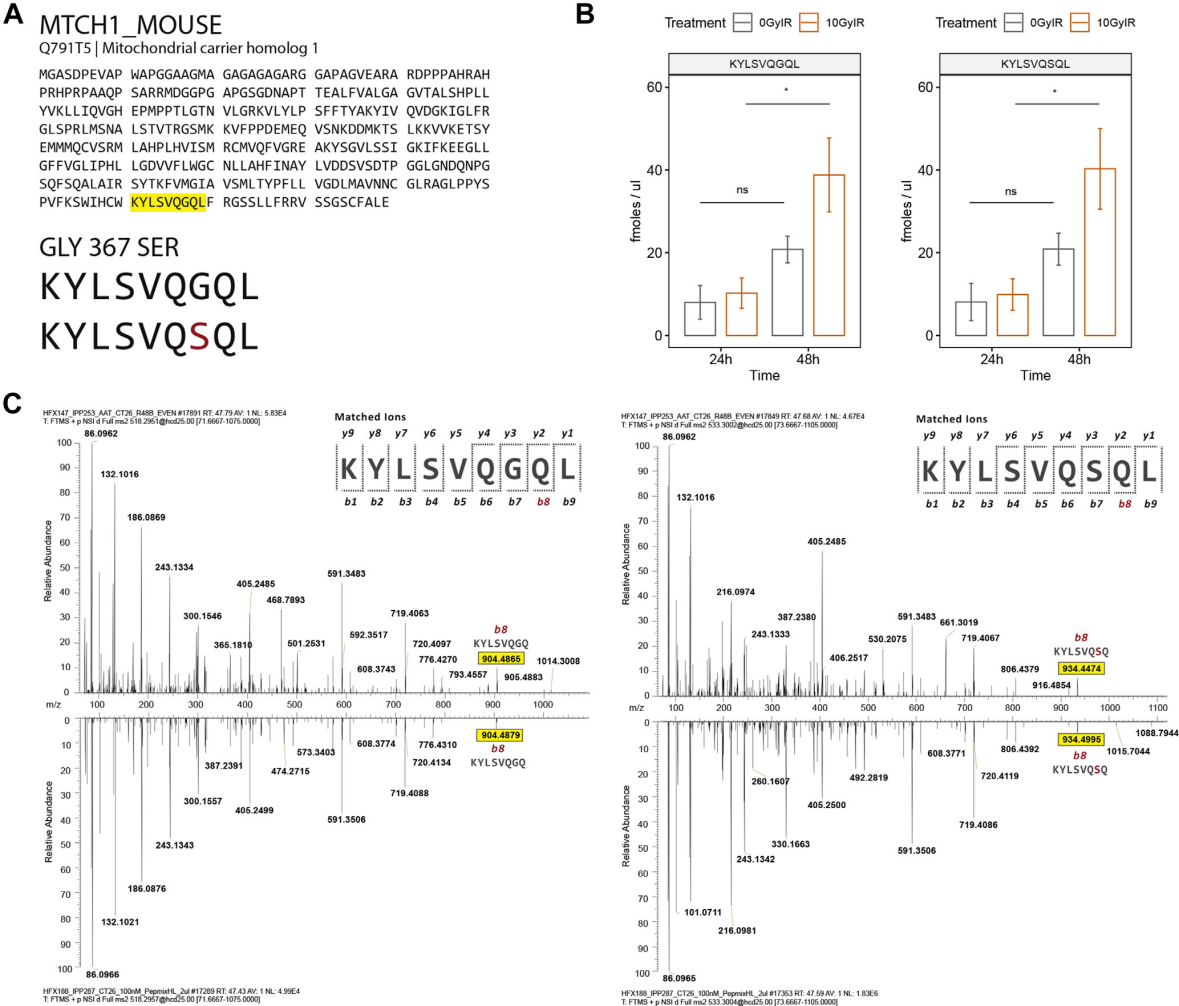
**Mass spectrometric validation of the KYLSVQSQL neoantigen.***A*, location of KYLSVQGQL and the G > S mutation in the MTCH1_MOUSE protein sequence. *B*, PRM-based absolute quantification of KYLSVQGQL and KYLSVQSQL peptides in the CT26 immunopeptidome at 24 and 48 h post 10 Gy irradiation. *C*, spectral mirroring of KYLSVQGQL (*left*) and KYLSVQSQL (*right*) of endogenous peptide found in the CT26 immunopeptidome at 48 h postirradiation against the synthetic spectrum. The b8 ion has been highlighted as the distinguishing ion between the unmutated and mutated peptide. *p*-values are representative of a paired student’s *t* test and have been denoted by ∗ <0.05 and ns for not significant. CT, cancer testis.

To assess whether irradiation has a direct effect on the presentation of neoantigens, we performed exome sequencing and mapped CT26-specific changes in comparison to the mouse reference genome, including the mapped mutations that led to alterations in protein sequences in the canonical mouse proteome. We identified a mutated MHC-peptide–derived from the Mitochondrial Carrier 1 protein MTCH1, which had a G > S mutation at position 367 to form a mutated variant KYLSVQSQL (Fig. 6*A*). To validate the observed increase in intensity of this neoantigen after irradiation, we performed a targeted PRM-based methodology including a synthetic, heavy labeled standard form of the peptide, modified at the N-terminal lysine residue, to measure an absolute concentration of this peptide in our samples (Fig. 6*B*). We estimated presentation of 103 peptide copies/cell in nontreated cells and 200 peptide copies/cell in irradiated cells, respectively (assuming 100% MHC peptide recovery), therefore indicating an approximate 2-fold increase in absolute amounts of MHC-associated peptide presented by irradiated cells *versus* controls. These experiments confirmed our previously observed trend of increasing copy number and intensity of the peptide with a significant increase at 24 and 48 h after treatment. The unmutated peptide sourced from the unaffected paired allele was also identified in the series which follows a similar trend. To confirm correct assignment of the peptide sequence, spectral matching of the unmutated and mutated peptide was performed to validate the difference in the single amino acid and to confirm the spectrum (Fig. 6*C*).

## Discussion

We provide a global proteomics and immunopeptidomics study of radiation-induced changes in both the cellular proteome and MHC-presented immunopeptidome in colorectal tumor cells. While confirming radiation-affected cellular pathways reported in the literature, we here expand the current knowledge of how radiation induces a modulation of the tumor immune visibility and demonstrate increased presentation of a neoantigen.

We observed upregulation of MHC class I molecules after radiation which has been previously described (4, 8). We here expand previous findings by presentation of an accurate quantitation of the upregulation of MHC class I in response to irradiation. We also observed differential regulation of MHC class I alleles; H-2-Dd with a maximal 3.25-fold increase, H-2-Kd with a maximal 2.59-fold increase, and H-2-Ld exhibiting a maximal 1.81-fold increase. This finding can be attributed partially to the relative changes in allele transcription which has shown to vary across alleles and has also previously been reported to change upon radiation (5). This result was reflected in the detected increase in peptide-repertoire associated to these three molecules in the immunopeptidomics analysis. After irradiation, both H-2-Dd and H-2-Kd had the largest peptide repertoire increase, while we detected only a smaller increase in H-2-Ld peptide repertoires when assigning the detected MHC peptides to their allele of origin by binding prediction. However, allele-specific biases can occur in mass spectrometric data as the chemical composition of peptides can alter their detection (32). For this reason, H-2-Ld, which contains a dominant proline as the primary P2 anchor, may offer in part, a reason for fewer peptides being detected.

We further detected changes in several upstream regulatory pathway of MHC class I induction, providing insight into the mechanism of MHC class I induction in response to radiation. The distinct changes we observed at 48 and 72 h after irradiation highlight the importance of incubation time in the determination of when to capture the ‘snapshot’ of the proteome or the immunopeptidome. A pathway-level exploration of the data showed strong support for the IFN-mediated upregulation of MHC, including several components of the interferon pathway and interferon response elements. These effects have been documented and validate our findings (33). Here, we detect an average 4.5-fold induction of STAT1 and STAT2 and confirm the upregulation of many of STAT-target genes, which have been previously reported as part of the IFN-induced gene signature including IFIT1, OAS1A, and ISG15 (34, 35). USP18 is also a regulator of the interferon signaling cascade, which is responsible for the cleavage of ISG15 that we found highly enriched in irradiated cells. The inhibition of this deubiquitinating enzyme can increase both radiosensitivity and can increase the effect of interferon signaling (36, 37).

We detected two cytosolic DNA sensing molecules that have been previously implicated in playing a role in the radiation induced IFN response in some cell types. ZBP1, which was significantly upregulated following radiation treatment, is a nucleic acid sensor that can bind to Z-DNA, a left-handed double helical structure which is less common than right-handed structures. Furthermore, ZBP1 is a key activator of necroptosis, initiating DNA leakage into the cytoplasm (38, 39). It has recently been shown that the ZBP1-MLKL necroptotic cascade induces cytoplasmic DNA accumulation in irradiated tumor cells and, in turn, autonomously activates cGAS-STING signaling, thus creating a positive feedback loop between these two pathways to drive persistent inflammation (40).

We also detected activation of the DDX58 (RIG-I) pathway as previously described (41, 42). Upon activation by cytoplasmic viral RNA sensing, it has been shown that cytosolic DNA can be used as a template for RNA polymerase III-driven synthesis of dsRNA, which can bind RIG-I and induce IFNβ production (43). DDX58 associates with the mitochondria antiviral signaling protein that in turn activates the IkappaB-related kinases TBK1 and IKBKE. These kinases then phosphorylate the interferon regulatory factors IRF3 and IRF7 which as a result translocate into the nucleus and transactivate IFN-alpha and IFN-beta interferons (42, 44). These regulatory factors had a low or negligible expression profile in our proteomic data, which was expected due to the exclusion of the nuclear proteome in our experimental preparation. DDX58 has been previously found to be highly expressed in mouse macrophages in response to irradiation but not in mouse melanoma (45). Through activation of an IFN response, DDX58 can also directly activate STING expression (46). Furthermore, radio-inducible proteins like DDX58 have been suggested as potential therapeutic targets (42). However, this has not been explored in the context of potential antigenic targets.

Immune evasion through the downregulation of MHC class I expression is a common feature to many cancers including colorectal cancer (47). Accordingly, the restoration of MHC expression on cancers has been proposed as potential treatment avenues through the induction of known MHC class I pathways, chemotherapy-induced expression, or through epigenetic silencing (48). The upregulation of type I interferons and the subsequent downstream effect of increased MHC class I expression in the context of radiation may offer a potential mechanism for treatment success of radiation therapy. This is further supported by documentation of an increased T cell infiltration after radiation therapy (49) and the requirement for both CD4 and CD8 T cells for successful treatment outcome (1, 50). MHC class I can be regulated by several pathways including the NFκB pathway, the activation of IRSE, and the more recently discovered NLRC5 pathway; the latter two are responsible for basal MHC class I levels (51). While NFκB itself showed little or no change in protein expression in our studies, IκBE expression differed at the 48 h time point indicating a possible decrease in the pathway’s inhibition (supplemental Fig. S7). A recent report proposed a STING-independent NLRC5 mechanism for the radiation-induced upregulation of MHC class I, but NLRC5 was not quantifiable within the depth of this study (8). Accordingly, it remains difficult to pinpoint a single mechanism by which the activation of interferons is taking place within this cell line. The activation of ZBP1 and RNA helicases DDX58 seem to point to an NFκB-mediated mechanism; however, the involvement of the cGas-Sting pathway is less clear (52, 53).

We have also profiled the upregulation of several components of the antigen presentation machinery including B2M, Tapasin, TAP1, and ERAP1 and all three classical MHC alpha chain variants present in this mouse strain, Kd, Dd, and Ld. The increase in ERAP1, although almost 2-fold at 72 h, could have a greater effect on the peptide repertoire at higher doses of radiation: ERAP1 is known to target the degradation of longer peptides to an optimal final minimal length of nine amino acids (54). However, we could here not observe a change in MHC class I peptide length following radiation treatment.

The validation of this data in a second cell line with a DNA damage deficiency, MC38, showed similar responses to the DNA damage competent cell line CT26 with the induction of interferons and antigen presentation being the most prevalent changes occurring in the proteome. Interestingly, these changes were less pronounced in the MC38 cell line at the proteome level but showed more significant changes at the 24 h time point in the immunopeptidome. Microsatellite unstable tumors are more responsive to immunotherapy and this is thought to be due to a higher mutational burden; however, in this analysis, it is worth noting that overall MHC peptide levels were much higher in the MC38 cell line than in CT26 which may increase opportunities for neoantigen expression. However, we would take some caution in making direct comparisons between the two cell lines as allele-specific mass spectrometric biases may contribute to the detection of MHC peptides (26).

Our profile of the CT26 immunopeptidome under the influence of radiation highlights a complex interplay between protein abundance and MHC antigen presentation, demonstrating that protein upregulation does not always result in higher levels of peptide presentation and vice versa. Turnover of proteins has a high correlation with antigen presentation, indicating that active protein degradation can drive antigen presentation in addition to increased translation (55). Our data also suggests that proteins may selectively undergo proteasomal degradation in the context of radiation and in return show increased presentation by MHC class I. Interestingly, the LIM protein AJUBA which has shown to play a role in augmenting tumor metastasis in colon cancer is strongly represented in this category (30). While there may be several factors which may contribute to the peptide presentation of endogenous proteins beyond protein turnover including protein length and the number of possible anchorable motifs present within that protein, the focus of this experiment was to understand the relationship between the differential expression of proteins and their associated change in representation in the immunopeptidome in response to radiation. Therefore, these comparative ratios represent the nuances of the relationship between protein expression and antigen presentation showing that several factors may contribute to this relationship.

It has been shown that radiotherapy and immune-checkpoint blockade induce systemic antitumor T cells in chemo-refractory metastatic non-small-cell lung cancer (56). In one patient, induced T cell responses could be defined as being specific toward a mutated tumor neoantigen, suggesting the importance of neoantigens for enhanced tumor immune recognition following radiation therapy. Recent studies have investigated prediction strategies based on potential mutated regions of genes to identify antigenic peptides upregulated in the context of radiation, including CAND1 and DHX58 gene, which were then tested for their efficiency as therapeutic vaccines in combination with radiotherapy (57). While these prediction strategies have shown some success, only selected neoantigen candidates have resulted in significantly enhanced immune recognition upon radiation. We have identified the increased presentation of a tumor neoantigen in response to radiation originating from MTCH1, KYLSVQSQL, containing tumor-specific mutation of glycine to serine at position 367.

In addition to the mutated peptide, we have identified a series of peptides originating from source proteins that were unique to the radiation condition, irrespective of their presence in the proteome. Peptides originating from unique pathways, in addition to several notable peptides have been identified. The protein of greatest interest was ADCY7, which had two representative peptides among others that were associated with catecholamine receptor signaling. This gene has previously been shown to be upregulated in gene expression studies by radiation (58). While individual peptides may play a role in discerning the mechanisms associated with radiation and antigen presentation, the possibility that these peptides can collectively contribute to an immune response should not be dismissed. Equally, the potential for alterations made to the tumor microenvironment to inflict changes to presented antigens should also be considered and could not be assessed in this study (59).

In this data set, no significant changes were observed with individual PTMs; however, a global increase in modifications at position 7 among 9-mer amino acids could augment T-cell recognition with an increased potential for steric contact at a site which is less often recognized compared to central residues like position 5 (60). A more robust study to target PTMs would be required to make conclusions from this preliminary analysis.

The efforts toward combinatorial therapies present a challenge to the field of antigen discovery and immunopeptidomics. The definition of radiation-induced antigens allows for the development of the next generation immunotherapies for a tailored combination therapy approach in cancer, which defines the focus of future work.

## Data Availability

The mass spectrometry proteomics data have been deposited to the ProteomeXchange Consortium *via* the PRIDE partner repository with the dataset identifier PXD032003 and 10.6019/PXD032003. The analysis of the absolute quantification has also been uploaded to Panorama Public at the following url: https://panoramaweb.org/ct26_mtch1_quant.url.

## Supplemental data

This article contains supplemental data

## Conflict of interest

The authors declare that they have no conflicts of interest with the contents of this article.
