# Supplementary material for: Ionizing Radiation Drives Key Regulators of Antigen Presentation and a Global Expansion of the Immunopeptidome
Source: Mol Cell Proteomics. 2022 Sep 9;21(11):100410. doi: 10.1016/j.mcpro.2022.100410 (PMC9579046; doi:10.1016/j.mcpro.2022.100410)
Supplement: Supplementary figures [file mmc1.pdf]

Ionizing radiation drives key regulators of antigen presentation and a global expansion of the immunopeptidome.

Supplementary Figures

Arun Tailor*[1,3], Hala Estephan[2], Robert Parker[1,3], Isaac Woodhouse[1], Majd Abdulghani[2], Annalisa Nicastri[1,3], Keaton Jones[4], Silvia Salatino[5], Ruth Muschel[2], Timothy Humphrey[2], Amato Giaccia[2], Nicola Ternette*[1,3]

[1]Centre for Immuno-Oncology, Nuffield Department of Medicine, University of Oxford, OX3 7DQ, UK

[2]Oxford Institute of Radiation Oncology, Department of Oncology, University of Oxford, OX3 7DQ, UK

[3]The Jenner Institute, University of Oxford, Oxford, OX3 7DQ, UK

[4]Nuffield Department of Surgical Sciences, John Radcliffe Hospital, Headington, Oxford, OX3 9DU, UK

[5]Wellcome Trust Centre for Human Genetics, Headington, Oxford, OX3 7BN, UK

*To whom correspondence should be addressed.

arun.tailor@ndm.ox.ac.uk

nicola.ternette@ndm.ox.ac.uk

Running Title: Radiation-induced antigen presentation.

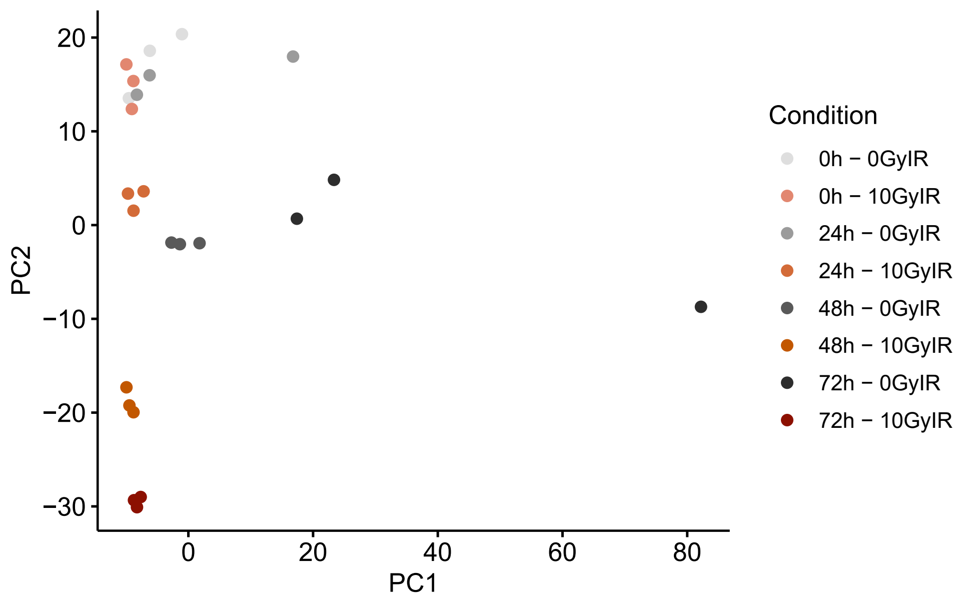

Fig S1 - PCA plot of treatment conditions and biological replicates. One outlier in the data (indicated by the arrow) has been plotted as an Inf value. Data is representative of three biological replicates at each time point encompassing two technical replicates which were averaged prior to analysis.

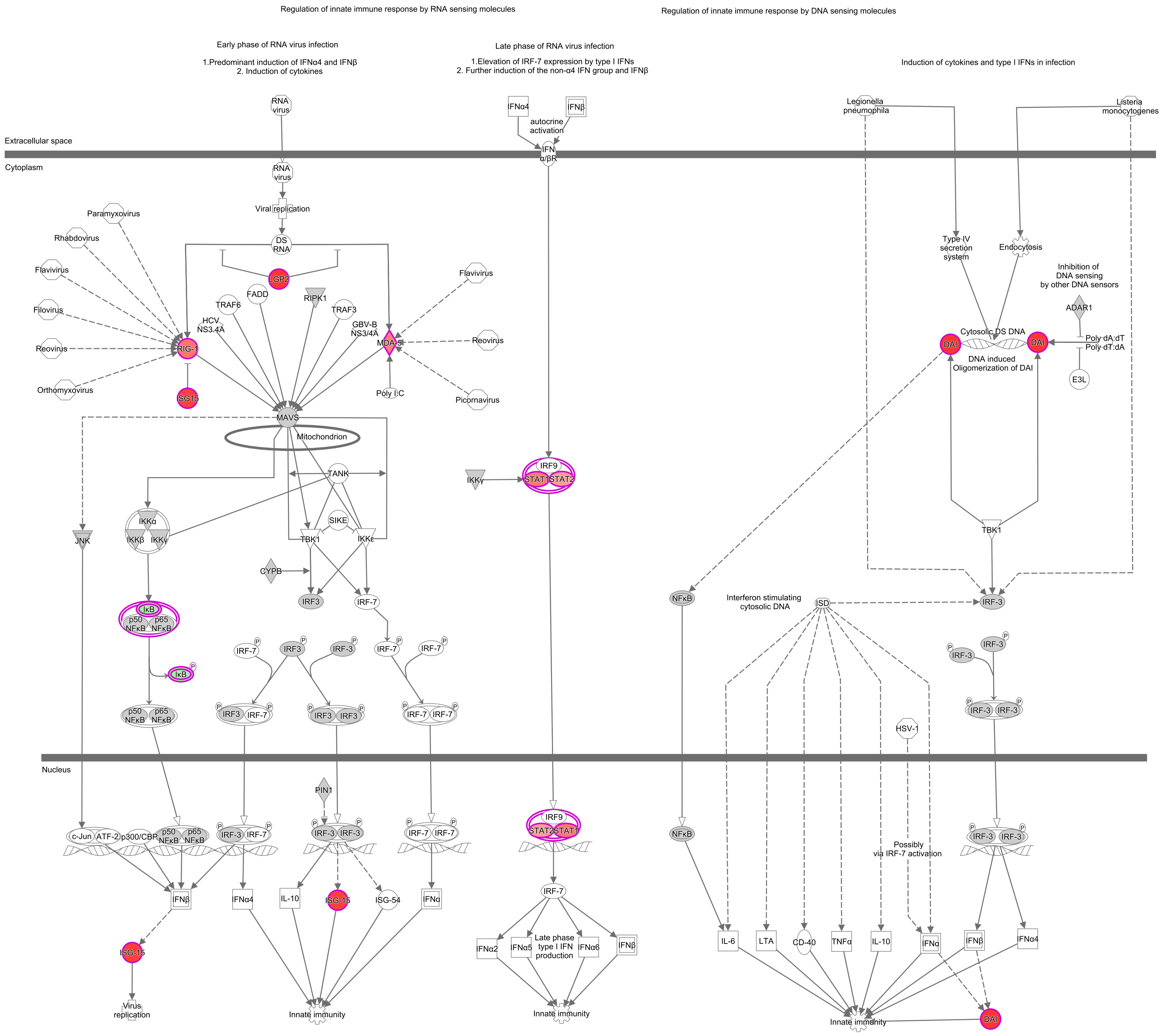

Fig S2 – Activation of IRF by Cytosolic Pattern Recognition Receptors Ingenuity Pathway visualized with P-values for quantifiable proteins in the CT26 proteome.

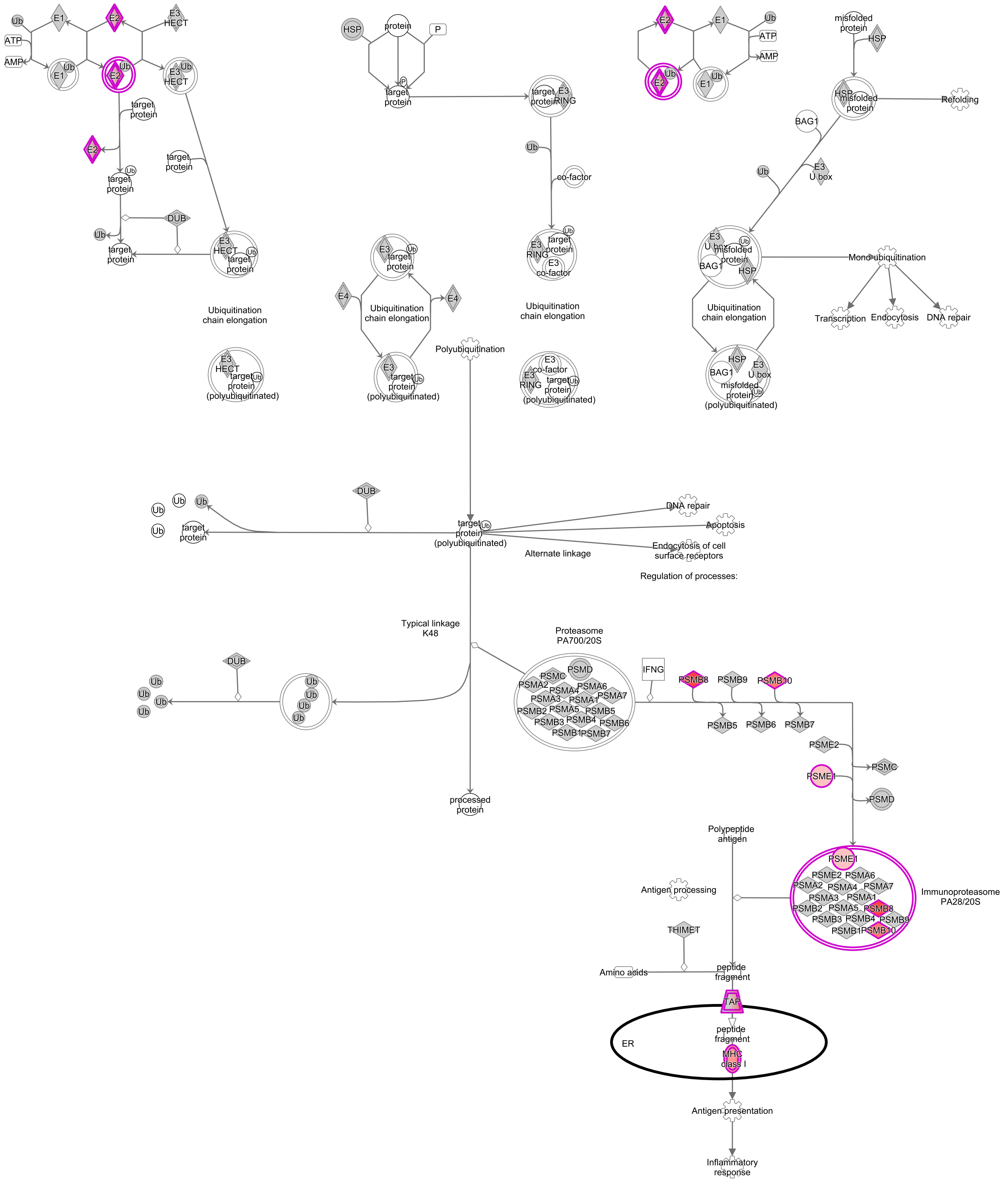

Fig S3 – Protein Ubiquitination Ingenuity Pathway visualized with P-values for quantifiable proteins in the CT26 proteome.

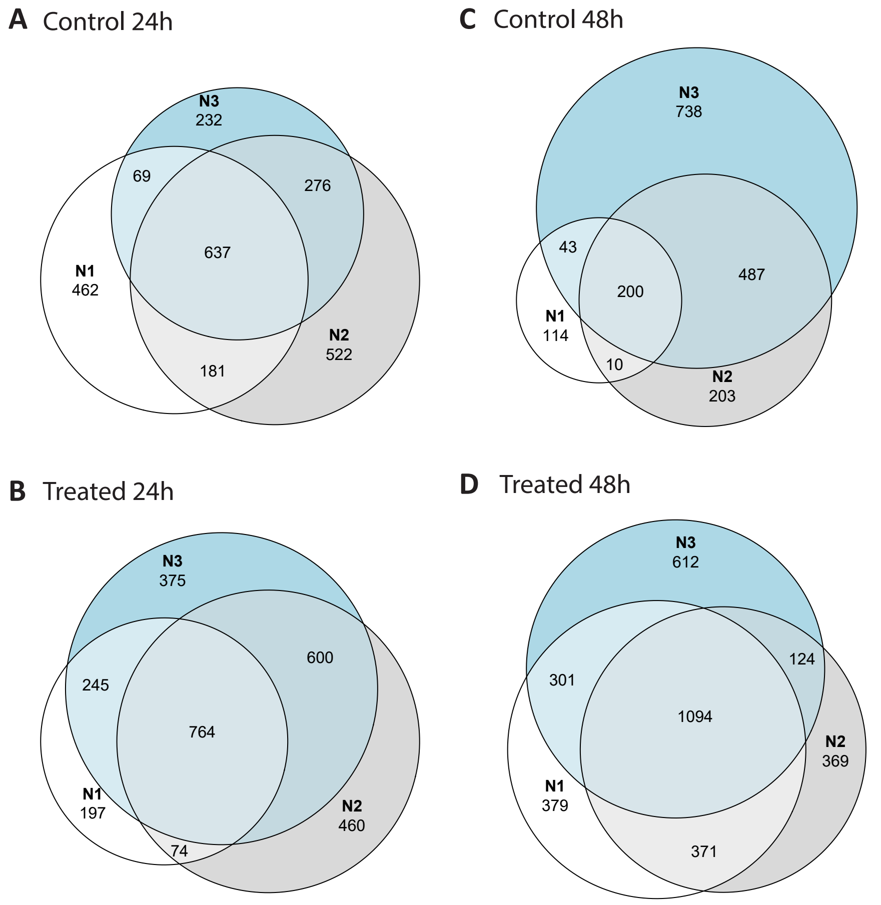

Fig S4 – Euler diagrams of overlap conditions of biological replicates of immunopeptidomics data. (A) CT26 0 Gy control at 24 hours (n=3). (B) CT26 10 Gy treated at 24 hours (n=3). (C) CT26 0 Gy control at 48 hours (n=3). (D) CT26 10 Gy treated at 48 hours (n=3).

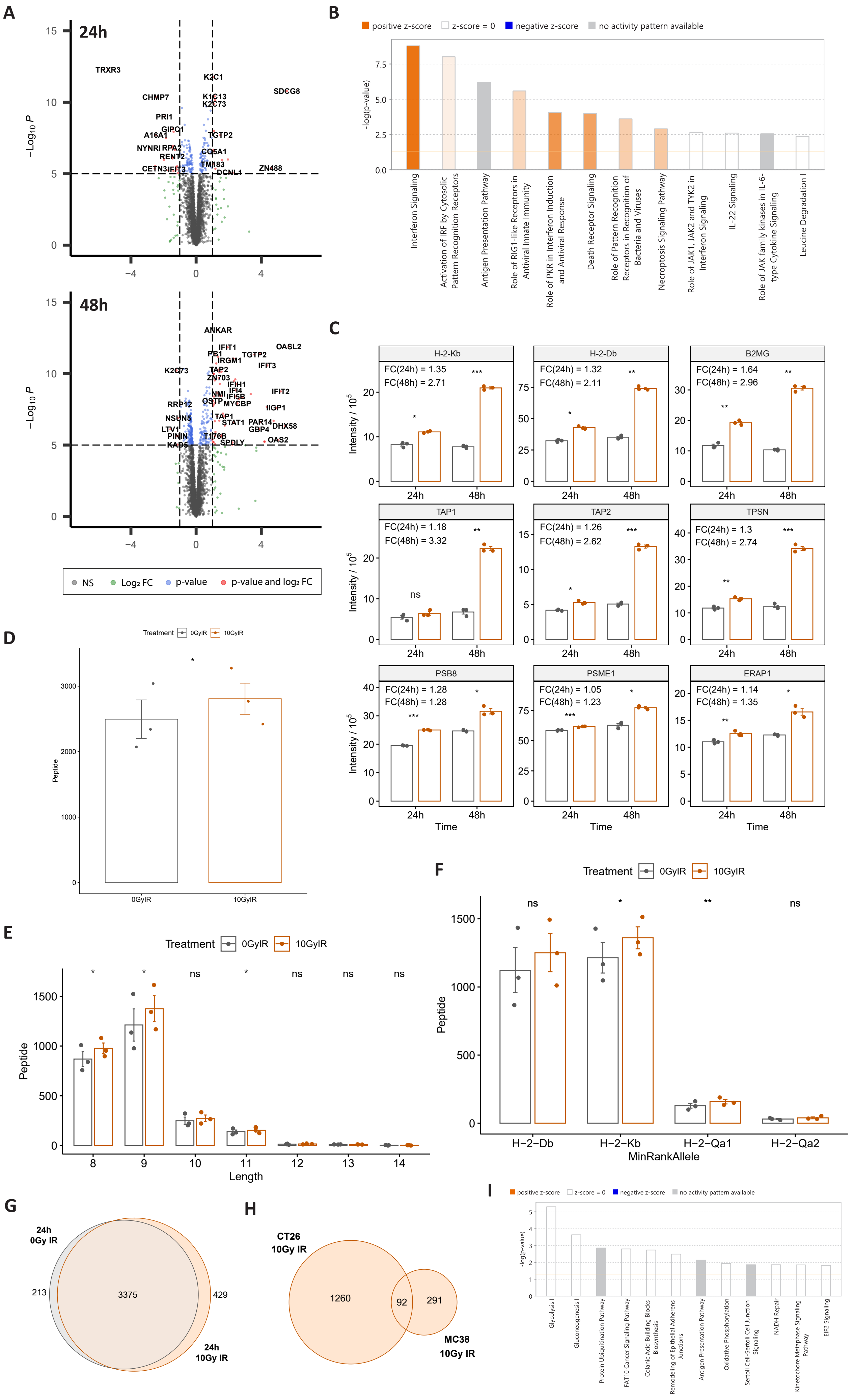
A
24h
TRXR3
CHMP7
PRI1
A16A1
GIPC1
NYNRI RPA2
RENT2
CETN3 IFIT3
K2C1
K1C13
K2C73
TGTP2
CO5A1
TM183
DCNL1
SDCG8
ZN488
48h
ANKAR
IFIT1
PB1
IRGM1
TGTP2
OASL2
IFIT3
K2C73
TAP2
ZN703
IFIH1
IFI4
NMI
IFI5B
IFIT2
OSTP
MYCBP
RRP12
IIGP1
NSUN5
TAP1
STAT1
PAR14
GBP4
DHX58
LTV1
PININ
T176B
KAP5
SPDLY
OAS2
$-Log_{10}$ P
NS
Log₂ FC
p-value
p-value and log₂ FC
B
positive z-score
z-score = 0
negative z-score
no activity pattern available
-log(p-value)
Interferon Signaling
Activation of IRF by Cytosolic Pattern Recognition Receptors
Antigen Presentation Pathway
Role of RIG1-like Receptors in Antiviral Innate Immunity
Role of PKR in Interferon Induction and Antiviral Response
Death Receptor Signaling
Role of Pattern Recognition Receptors in Recognition of Bacteria and Viruses
Necroptosis Signaling Pathway
Role of JAK1, JAK2 and TYK2 in Interferon Signaling
IL-22 Signaling
Role of JAK family kinases in IL-6-type Cytokine Signaling
Leucine Degradation I
C
H-2-Kb
FC(24h) = 1.35
FC(48h) = 2.71
H-2-Db
FC(24h) = 1.32
FC(48h) = 2.11
B2MG
FC(24h) = 1.64
FC(48h) = 2.96
TAP1
FC(24h) = 1.18
FC(48h) = 3.32
TAP2
FC(24h) = 1.26
FC(48h) = 2.62
TPSN
FC(24h) = 1.3
FC(48h) = 2.74
PSB8
FC(24h) = 1.28
FC(48h) = 1.28
PSME1
FC(24h) = 1.05
FC(48h) = 1.23
ERAP1
FC(24h) = 1.14
FC(48h) = 1.35
Intensity / 10^5
Time
24h
48h
D
Treatment 0GyIR 10GyIR
Peptide
0GyIR
10GyIR
E
Treatment 0GyIR 10GyIR
Peptide
Length
F
Treatment 0GyIR 10GyIR
Peptide
H−2−Db
H−2−Kb
H−2−Qa1
H−2−Qa2
MinRankAllele
G
24h
0Gy IR
213
3375
429
24h
10Gy IR
H
CT26
10Gy IR
1260
92
291
MC38
10Gy IR
I
positive z-score
z-score = 0
negative z-score
no activity pattern available
-log(p-value)
Glycolysis I
Gluconeogenesis I
Protein Ubiquitination Pathway
FAT10 Cancer Signaling Pathway
Colanic Acid Building Blocks Biosynthesis
Remodeling of Epithelial Adherens Junctions
Antigen Presentation Pathway
Oxidative Phosphorylation
Sertoli Cell-Sertoli Cell Junction Signaling
NADH Repair
Kinetochore Metaphase Signaling Pathway
EIF2 Signaling

Fig S5 – Proteomics and immunopeptidomics analysis of radiation on the MC38 cell line. (A) Volcano plots of differentially expressed proteins (red) isolated by time point (limma, -Log10P > 5, -Log2 fold change > 1.5). (B) Ingenuity Pathway Analysis of differentially expressed proteins at 48h post 10 Gy irradiation (-Log2 fold change > 1.5). (C) Normalized intensity plots of proteins encompassing the Antigen Presentation Pathway in the MC38 proteome upon irradiation. Points are representative of Mean ± SD values of three biological replicates and P-values are representative of a two-way anova, where grey indicates untreated, and orange indicates 10 Gy IR treated. (D) Total MHC peptide intensity at 0 Gy IR and 10 Gy IR at 24 hours post irradiation. (E) Length intensity distribution of MHC peptides at 0 Gy IR and 10 Gy IR at 24 hours post irradiation. (F) Allele binding intensity distribution at 0 Gy IR and 10 Gy IR at 24 and 48 hours post irradiation. (G) Unique MHC peptides at 0 Gy IR and 10 Gy IR at 24 hours post irradiation. (H) Overlap of source proteins between MC38 and CT26 radiation-specific peptides. (I) Ingenuity Pathway Analysis of overexpressed source proteins derived from overlap between MC38 and CT26 radiation-specific peptides. P-values for the immunopeptidomics data (D-F) are representative of a paired student's t-test and have been denoted by * < 0.05, ** < 0.01, *** < 0.001 and ns for not significant.

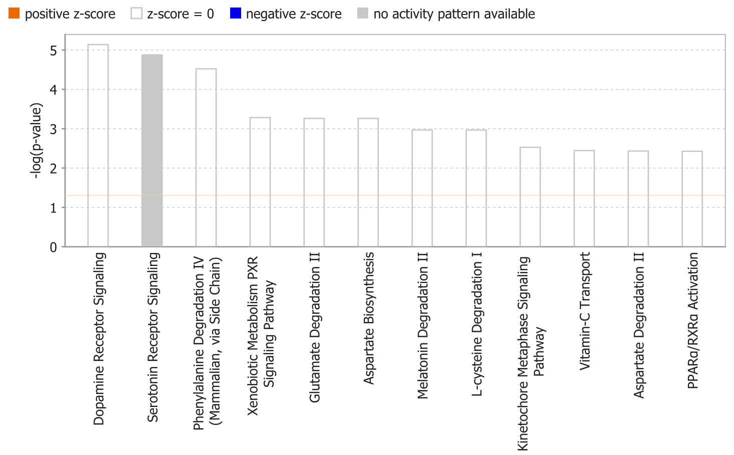

Fig S6 – Ingenuity Pathway Analysis of overexpressed source proteins derived from filtered radiation-specific peptides designated against expanded CT26 control data.

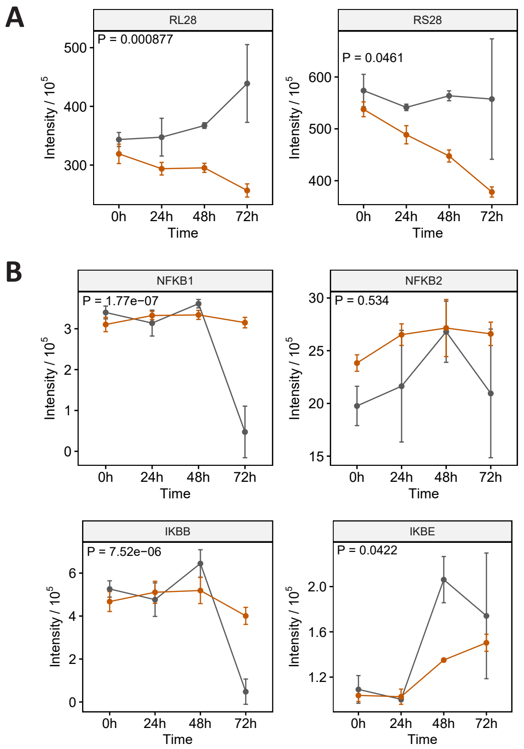

Fig S7 - Intensity plots of select proteins encompassing various pathways upon irradiation. (A) RS28 & RL28 Ribosomal Proteins. (B) NFKB subunits. Points are representative of Mean ± SD values of three biological replicates and P-values are representative of a two-way anova, where grey indicates untreated, and orange indicates 10 Gy IR treated.

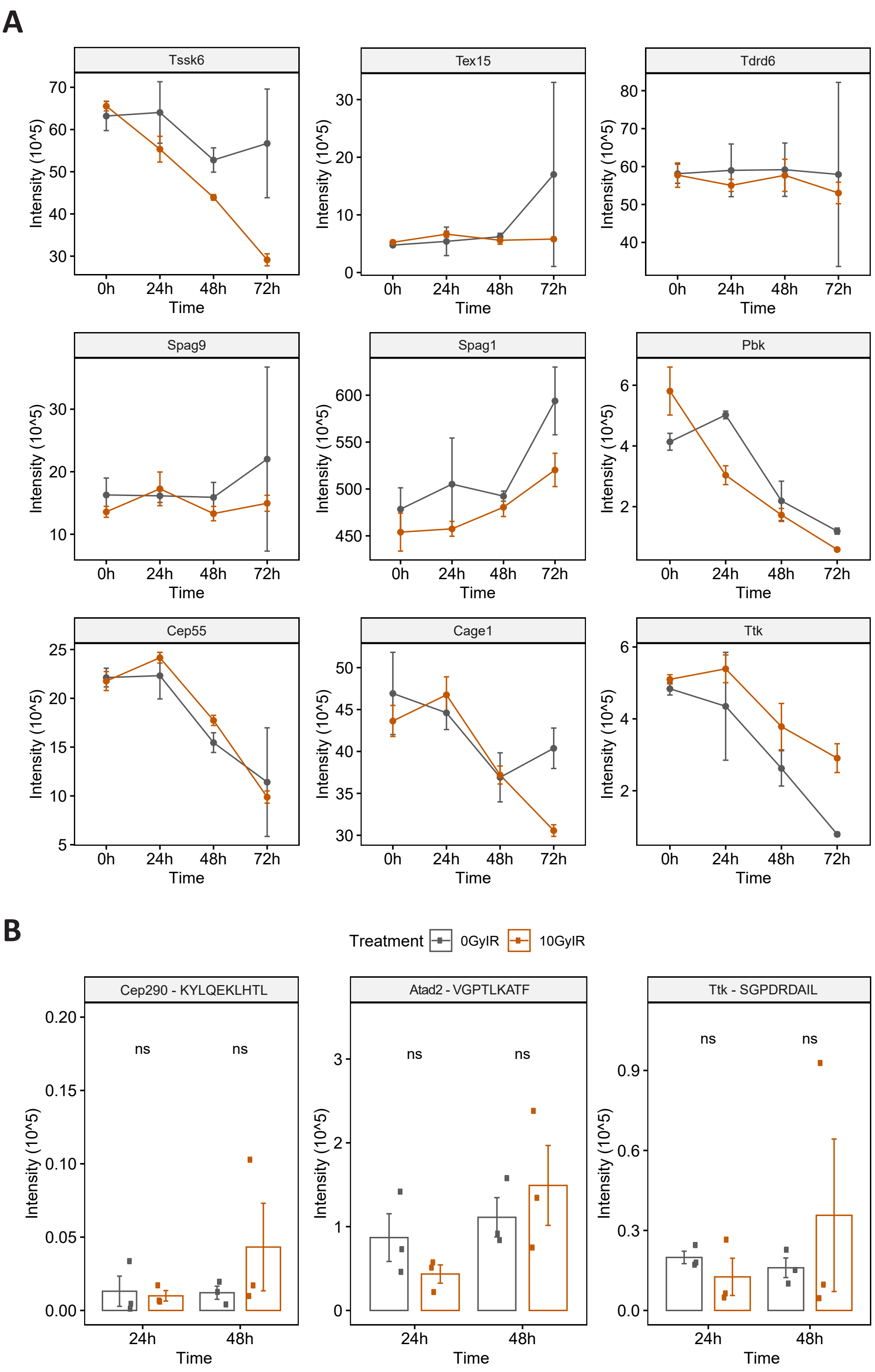

Fig S8 – Exploration of CT antigen matches in proteomics and immunopeptidomics data. (A) Intensity plots of matched CT antigens identified in the CT26 proteome. Points are representative of Mean ± SD values of three biological replicates. (B) Bar charts of peptide intensities of matched CT antigens identified in the CT26 immunopeptidome. Points are representative of Mean ± SD values of three biological replicates are representative of a paired t-test, where grey indicates untreated, and orange indicates 10 Gy IR treated. P-values for the peptide data are representative of a paired student's t-test and have been denoted by * < 0.05, ** < 0.01, *** < 0.001 and ns for not significant.

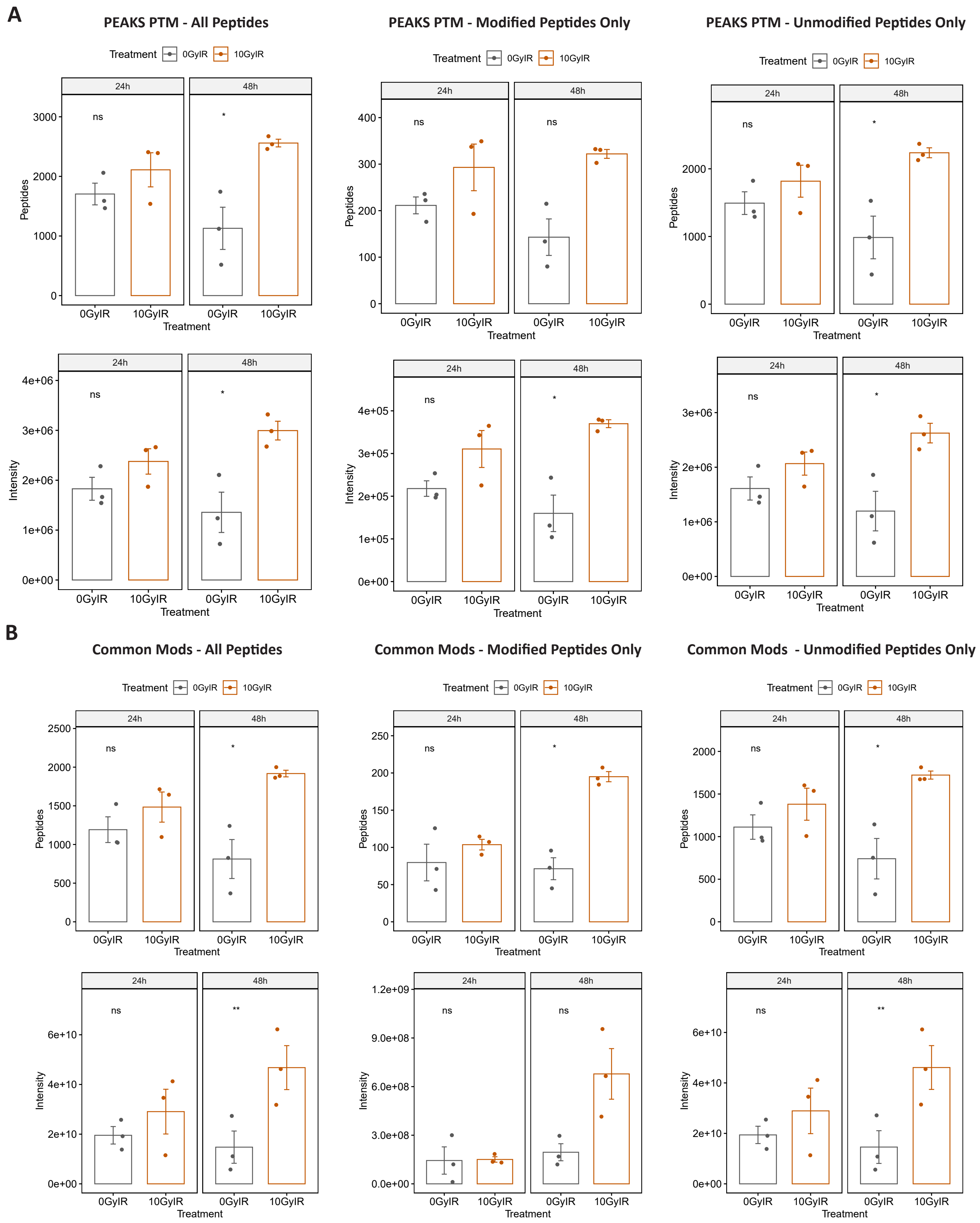

Fig S9 – Assessment of PTM search influence on radiation-specific changes to CT26 immunopeptidome. (A) Analysis of immunopeptidome following search of data using PEAKS PTM method. (B) Analysis of immuno-peptidome following search of data including common variable modifications: NQ deamidation, cysteinylation, methionine oxidation and protein n-terminal acetylation. All peptides include total peptides identified and summed peptide intensity at 24 and 48 hours post 10 Gy irradiation. Modified peptides include filtered sidentified and summed modified peptide intensity at 24 and 48 hours post 10 Gy irradiation. Unmodified peptides include identified and summed unmodified peptide intensity at 24 and 48 hours post 10 Gy irradiation. P-values are representative of a paired student's t-test and have been denoted by * < 0.05, ** < 0.01, *** < 0.001 and ns for not significant.

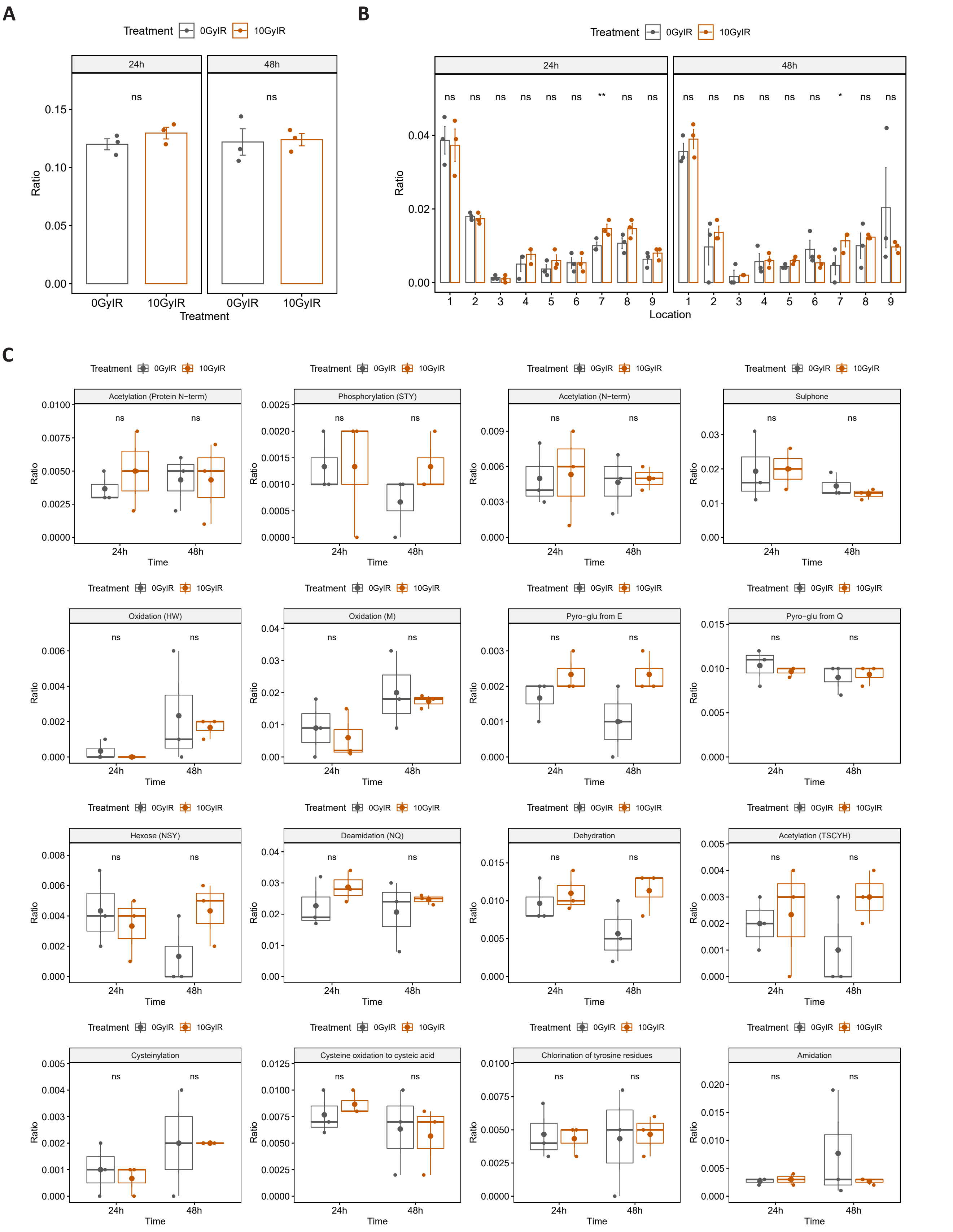

Fig S10 – Analysis of global PTM changes on the CT26 immunopeptidome upon radiation treatment. (A) Total ratio of modified peptides against unmodified peptides at 24 and 48 hours post 10 Gy irradiation. (B) Ratio of modified peptides against unmodified peptides at 24 and 48 hours post 10 Gy irradiation at each amino acid in 9-mer peptides. (C) Ratio of individual modifications on peptides against unmodified peptides at 24 and 48 hours post 10 Gy irradiation. P-values are representative of a paired student's t-test and have been denoted by * < 0.05, ** < 0.01, *** < 0.001 and ns for not significant.
